# Should We Cheer Together? Gender Differences in Instantaneous Well-being: An Application to COVID-19 Lockdowns

**DOI:** 10.1007/s10902-022-00574-7

**Published:** 2022-12-07

**Authors:** José Ignacio Giménez-Nadal, José Alberto Molina, Jorge Velilla

**Affiliations:** 1 Department of Economic Analysis, University of Zaragoza, IEDIS, C/ Gran Vía 2, 50005 Zaragoza, Spain; 2 GLO, Global Labor Organization, Maastricht, The Netherlands

**Keywords:** Subjective Well-Being, Togetherness, Gender Difference, D10, J16, J22

## Abstract

The COVID-19 pandemic has confined millions in their homes, an unprecedented opportunity to spend more time together with family members. This paper explores subjective well-being in the uses of time for US and UK workers, differentiating between solo activities and activities done with family members, at home and outside the home. Using American and British time use surveys, we compute the instant utility associated with paid work, unpaid work, leisure, and childcare activities. OLS regressions on both men and women show that workers prefer joint leisure to solo leisure, and that significant differences exist for solo and joint market work and housework, between the sexes. Despite that, the effect magnitudes are relatively low. Furthermore, we simulate a strict lockdown situation by replacing where and with whom worker episodes would be, based on mid-2020 strict confinements. Results suggest diverging effects, since more time with the spouse/partner and children, and less time with others, seems to increase the experienced wellbeing of women, compared to that of men. The simulation exercise also reveals asymmetric effects in the US and in the UK. The conclusions of this paper may help in assessing the psychological consequences of COVID-19 lockdowns, beyond the negative economic and labour market consequences.

## Introduction

The COVID-19 pandemic spread across the world, with serious consequences for daily life, including the confinement of individuals in their homes. This is not to trivialize the devastating death toll, nor the unprecedented damage to the global economy, but this confinement has clear implications for the time-allocation decisions of families, as many parents are forced to telework, and to take care of their children, with no in-person school classes (Boca et al., 2020; Sevilla & Smith, 2020). Thus, the time spent with spouses/partners, children, and other family members is certain to increase as a consequence of the confinement. This, in turn, has significant implications for individual well-being, as time spent with others is generally preferable to solitude (Sullivan, 1996a, b; Hallberg, 2003; Kahneman et al., 2004; Jenkins & Osberg, 2004; Sevilla et al., 2012; Cosaert et al., 2022), and loneliness may lead to lower well-being (Hamermesh, 2020).

Prior research has analyzed the link between time-allocation decisions and affective well-being (Kahneman et al., 2004; Kahneman & Krueger, 2006; Krueger, 2007; Sevilla et al., 2012; Gimenez-Nadal & Molina, 2015; Gimenez-Nadal et al., 2020).^1^ For instance, Kahneman et al., (2004) find that leisure activities are superior in terms of instant enjoyment to activities such as commuting, market work, or housework. Gimenez-Nadal & Molina (2015) find that voluntary activities produce enjoyment for the participants in those activities, with a spillover effect on other daily activities. The research in this field has also shown that spending time with others is preferable to solitude (Sullivan, 1996a; Kahneman et al., 2004; Helliwell & Putnam, 2005; Sevilla et al., 2012). For instance, Sullivan (1996a) finds that time spent in different activities is more enjoyable when spent in company, in comparison to being alone. Kahneman et al., (2004) find that activities done in the presence of friends, relatives, and the spouse and children are superior in terms of utility, compared to doing something alone. Sevilla et al., (2012) reach the same conclusions, although restricting their analysis to leisure activities.

If the COVID19 pandemic and its associated confinements and lockdowns are related to changes in the time devoted to activities and with whom those activities are done, it is important to examine the consequences of these confinements and lockdowns in terms of time-allocation decisions, togetherness, and well-being, to understand the impact of confinement on individual daily life, and on gender differences in well-being. While some studies have found that confinements and lockdowns have led many couples to regress towards more traditional gender roles (Boca et al., 2020; Sevilla & Smith, 2020), others have reported a more equal division of labor between men and women (Boll et al., 2021; Sevilla & Smith, 2020). Thus, gender differences in the changes in affective well-being may arise from COVID19 measures, since more leisure may represent increased affective well-being, more housework may do the opposite. Gender differences in the instant enjoyment obtained from similar activities have been reported, with women reporting higher affective well-being during leisure and housework activities (Krueger, 2007; Sevilla et al., 2012; Gershuny, 2013; Qian & Fan, 2019).

Within this framework, this paper analyzes the experienced utility (or instantaneous well-being, as in Kahneman et al., 2004) of workers in the United Kingdom and the United States, using the UK Time Use Survey (UKTUS) 2014–2015, and the American Time Use Survey (ATUS) 2010–2013, and focusing on the difference between activities done alone and those in the presence of other household members.^2^ We find that women benefit more from the presence of others in their daily activities, as the increase in experienced utility when the activity is done in the presence of others, in comparison to being alone, is greater for women than for men. This gender difference, in line with the findings of Qian & Fan (2019), is limited to market work and housework activities, since men and women both experience similar increases in their experienced utility when leisure happens in the presence of others.^3^

We also simulate a lockdown, by assuming changes in individual time allocations (e.g., less paid work time, and more leisure time), where such activities take place (at home), and with whom they are done (alone, with the spouse, with children, and/or other relatives). The simulation is based on a strict lockdown in which all activities are done at home, alone for individuals who live alone, or together with the respective cohabitants (e.g., a partner, children, and/or others) for individuals who cohabit with others. The changes in the experienced utility of individuals under the simulation, compared to the general setting, reveal asymmetric effects for the US and the UK, as the lockdown has different impacts in these countries, in addition to different effects for women and men. Overall, confinement has negative effects on experienced enjoyment among UK individuals, but positive effects for US individuals.

The gender and country analysis of experienced utility may help in understanding the possible consequences of confinement caused by the COVID-19 pandemic, beyond the negative consequences on both the economy and the labour market. Women appear more likely to do things alone than men, and to the extent that confinement may mean more time with family members, the confinement itself may imply greater increases in well-being for women. However, the gains from more time with family members may be outweighed by the fact that the COVID-19 outbreak has amplified the need for care work within the home, not only due to school closures, but also due to the large number of individuals contracting the virus and being quarantined. In a world where women do relatively more unpaid care work than do men (Eaton, 2005; Carmichael et al., 2008), this pandemic may have increased the demands on women’s time and thus increased the gender imbalance of housework (including care work) time (Aguiar & Hurst, 2007; Gimenez-Nadal & Sevilla, 2012).

Furthermore, when individuals are at home as a consequence of job losses, there is a gender asymmetry in how time allocations are redistributed; while women increase the time they devote to housework, men increase the time devoted to personal care and leisure (Beblo & Robledo, 2008; Aguiar et al., 2013; Berik and Kongar, 2013; Gimenez-Nadal & Molina 2014; Boca et al., 2020; Sevilla & Smith, 2020). The gender asymmetry in the gains in utility from more time with family members, favoring women, may compensate for the negative consequences of the extra workload for women.

Our contribution to the literature is twofold. We first contribute to the analysis of gender differences in experienced utility, with a focus on how the presence of others during the different activities is related to changes in the experienced utility of men and women. Prior research has analyzed this for a range of activities, finding a positive relationship (Sullivan, 1996a; Kahneman et al., 2004; Sevilla et al., 2012). However, this prior research has not explored gender differences in the positive relationship. We find that, in general terms, the increase in experienced utility when the activity is done in the presence of others, in comparison to being alone, is greater for women than for men. Second, we contribute to the literature by analyzing the effects of COVID19 confinements and lockdowns on individual well-being (Brand et al., 2020; Foa et al., 2020; Recchi et al., 2020; Brindal et al., 2021; Fujiwara et al., 2020; Long, 2021; Möhring et al., 2021; Ruiz et al., 2021; Zacher & Rudolph, 2021). The evidence presented in these studies is far from conclusive, and is based on cognitive, subjective well-being measures. We use an alternative approach, with a simulation and analysis of affective experienced utility while doing activities, as an instrument to evaluate possible gender differences in such effects.

## Background

The analysis of the effects of the COVID-19 pandemic - and the confinements and lockdowns that followed - on individual subjective well-being has received special attention in recent months, generating a flourishing field of research. Some authors have conducted specific research based on pilot analyses or specific surveys to study how lockdowns have affected daily behaviors and well-being, although there is no consensus on the effects of confinements and lockdowns on subjective well-being.^4^ Recchi et al., (2020) reported increased well-being derived from lockdowns in France, and Foa et al., (2020) found that lockdowns reduced individual negative effects in the UK, thus increasing subjective well-being. Similarly, Long (2021) found increased subjective well-being of US individuals during the US lockdown, and Brand et al., (2020) found that physical exercise during lockdowns had a positive impact.

Other authors have reported decreased well-being during the COVID-19 lockdowns. Möhring et al., (2021) reported declines in family and work satisfaction in Germany during strict lockdowns, while Zacher & Rudolph (2021) found no differences in subjective well-being in the same country. Ruiz et al., (2021) found different channels through which COVID-19 affected well-being negatively, such as physical health in the UK, and emotional well-being in Latin American countries. In Australia, Brindal et al., (2021) found that lockdowns generated increased isolation, leading to decreases in well-being (although using a non-representative online survey). A recent report on the negative implications of COVID-19 on well-being, with a focus on the UK, is provided by Fujiwara et al., (2020). Hamermesh (2020) studied how loneliness and togetherness correlate with overall cognitive well-being (i.e., overall life satisfaction), finding that loneliness correlates with decreased satisfaction for both singles and couples, in general terms.

Prior research is based on respondents’ subjective well-being by asking for general satisfaction with life, or general happiness with life, which aims to capture the subjective cognitive evaluations (subjective well-being measures often referred to as Satisfaction With Life Scale, SWLS).^5^ Alternative approaches to measure the well-being of individuals exist, including measures aimed at capturing the affective component of subjective well-being (e.g., affective well-being); that is to say, the subjective evaluation of emotions experienced during one’s daily life (Watson et al., 1988). A popular tool to measure the affective component of well-being is the Positive Affect Negative Affect Scale (PANAS), which measures individuals’ experience of positive and negative affect. The measurement of affective well-being linked to time-allocation decisions is known as instantaneous well-being, or experienced utility (Kahneman et al., 2004).

There are several methodologies to assess the link between activities and instant feelings, including the use of *Activity Enjoyment Ratings* (Juster & Stafford, 1985; Gershuny & Halpin, 1996) and the *Experience Sampling Method*. Alternative methods of collecting affective data on hedonic experience, such as the conventional *yesterday diary* used in time-budget surveys (Szalai, 1972) and the *Day Reconstruction Method* (Kahneman et al., 2004), are less costly to implement. Both methods collect information on how the respondent experienced all or some of the activities he/she engaged in during the previous day, as described in a time-use diary. This approach has been used to measure national well-being (Krueger, 2009; Stiglitz, Sen and Fittousi, 2009; Gershuny 2013), and individual daily happiness (Kahneman et al., 2004; Kahneman & Krueger, 2006; Krueger, 2007; Knabe et al., 2010; Sevilla et al., 2012; Gimenez-Nadal & Molina, 2015; Hoang & Knabe, 2021).

Several authors have shown that daily activities are linked to the well-being of individuals, defined in terms of instant enjoyment, experienced utility, or positive/negative affect (Kahneman et al., 2004; Kahneman & Krueger, 2006; Krueger, 2007; Sevilla et al., 2012; Gimenez-Nadal & Molina, 2015; Gimenez-Nadal et al., 2020).^6^ For instance, Kahneman et al., (2004) find that leisure activities are superior in terms of instant enjoyment to activities such as commuting, market work, or housework. Gimenez-Nadal & Molina (2015) find that voluntary activities produce enjoyment for the participants, with a spillover effect on the rest of daily activities. Research in this field has also shown that spending time with others is preferable to solitude, as there is evidence from instant enjoyment data suggesting that individuals report higher levels of instant enjoyment from activities done in the company of others than those done alone (Helliwell & Putnam, 2005; Kahneman et al., 2004). For instance, Sullivan (1996a) analyzes time use in couples in the UK, finding that time spent in different activities is more enjoyable when it is spent in company. Kahneman et al., (2004), using data on experienced utility for a sample of 909 working women in the US, found that activities done in the presence of friends, relatives, and the spouse and children are superior in terms of utility, compared to acting alone. Sevilla et al., (2012) find that, for both the United Kingdom and the United States, the presence of young children is associated with greater happiness.

Given that confinements and lockdowns derived from the COVID19 pandemic may lead to changes in the allocation of time, and the presence of others during time use activities may have implications for individual well-being. Two contrasting hypotheses about the impact of the pandemic on gender divisions of unpaid work have emerged. The “re-traditionalization hypothesis” claims that the pandemic is a ‘patriarchal pandemic’ (Kreyenfeld & Zinn, 2021). More concretely, it is assumed that the pandemic and accompanying measures has led many couples to regress towards more traditional gender roles, whereby women, and in particular mothers, are more likely to shoulder additional housework and childcare responsibilities, almost exclusively (Boca et al., 2020; Sevilla & Smith, 2020). In contrast, the “equalizing hypothesis” claims that the COVID-19 crisis may have promoted a more equal division of labor between men and women in some families (Boll et al., 2021; Sevilla & Smith, 2020). This latter hypothesis is based on women’s over-representation in essential occupations, and hence their crucial role in society’s pandemic response. On the other hand, as the pandemic has forced many workers into short-time work and working from home, men and fathers may have had more time at their disposal or may have spent more time at home. In combination with a greater exposure to family life and the possible inability of the woman to take on the additional workload, men’s and in particular fathers’ involvement in childcare and household tasks may have increased (Yerkes et al., 2020; Sevilla & Smith, 2020; Carlson et al., 2021).

Despite that an increasing body of research has found changes in time allocation derived from confinements, the results are somewhat inconclusive. Some evidence points to increased engagement of mothers in unpaid work (Boca et al., 2020; Farré et al., 2020), although other studies show that men, and particularly fathers, have become more involved in unpaid work, thus narrowing the gender gap (Craig and Churchill, 2021; Carlson et al., 2021; Yaish et al., 2021). These diverse and sometimes contradictory results are related to a range of factors, such as the exact population under study, the country’s cultural context, and its pandemic response, as well as the type of data used. Thus, the possible effects of COVID-19 confinements on affective well-being, measured through instant enjoyment of activities, are not clear, nor are the differential effects depending on gender.

## Data and Variables

We use diary data from the UK Time Use Survey (UKTUS), for the years 2014–2015, and the Subjective Well-being (SWB) module of the American Time Use Survey (ATUS) for the years 2010, 2012 and 2013.^7^ Apart from providing information on the socio-demographic characteristics of respondents, these surveys include time use diaries, with information on respondents’ activities during the 24h of the day, from 4 am to 4 am of the following day. Time use diaries have become a common tool in analyzing individual time allocation and daily behaviors, as they produce more reliable estimates than surveys based on stylized questionnaires. The UKTUS is the official time use survey of the UK and is sponsored by the Centre for Time Use Research, while the ATUS is the official time use survey of the US, and is conducted by the US Bureau of Labor Statistics. The ATUS Well-being modules were fielded from January through December in each year, and the 2015 UKTUS was fielded from April 2014 through March 2015.

The time use categories analyzed are based on Aguiar & Hurst (2007) and Gimenez-Nadal & Sevilla (2012), and we define paid work, unpaid work, childcare, and leisure. Paid work includes those activities related to employment, excluding commuting. Unpaid work time is defined as those activities related to household chores and domestic activities (cooking, setting the table, washing, cleaning, adult care).^8^ Childcare time includes all activities related to the care of children, and includes basic, educational, and supervisory childcare. For leisure time, we consider activities such as watching TV, sports, out-of-home leisure, gardening, pet care, and socializing.

The UKTUS and the SWB module of the ATUS include information at the diary level on the feelings experienced by individuals during their daily episodes. Both surveys use the Day Reconstruction Method, in which respondents are asked to fill out a diary summarizing episodes of activities for the selected day, and then are asked about their feelings during the activities (Kahneman et al., 2004).^9^ In the ATUS SWB module, respondents are asked to rank three randomly selected episodes, lasting at least five minutes, describing the extent to which they were happy, stressed, sad, tired, or felt pain. Values are recorded on a 7-point scale, with “0” indicating that the respondent “did not experience the feeling at all”, and “6” indicating that a “feeling was extremely strong”. In the UKTUS, respondents answered the question “How much did you enjoy this time?”, with possible answers going from 1 “not at all” to 7 “very much”. Compared to the ATUS SWB module, the UKTUS collects instantaneous well-being information for all episodes in the diary. Although the type of questions used to elicit a respondent’s instantaneous well-being differs between the UK (enjoyment) and the US (happiness) surveys, research suggests that the two types of measure are highly correlated (Knabe et al., 2010).^10^ The type of well-being that can be measured with the ATUS Well-Being Module and the UK Time Use Survey refers to the instantaneous affective well-being, experienced utility, or instant feelings experienced by individuals throughout the day.

We have computed the coefficients for the happiness and enjoyment scales, to determine whether they are reliable and consistent concerning the respondents. We have estimated the $\alpha$ and $\omega$ reliability coefficients, which are about 0.746 and 0.738 for the UK, and 0.753 and 0.777 for the US. These measures are above the standard threshold for research purposes, often fixed at 0.7 (Cortina, 1993), and rank as “high” or “fairly high” (Taber, 2018), suggesting that they are consistent and the analysis is appropriate. We have also computed the average inter-item covariances, which are found to be 0.511 for the UK enjoyment scale, and 1.198 for the US happiness scale, along with the inter-class correlation between averages on the same individuals, which are 0.575 in the UK sample, and 0.774 in the US sample. In both cases, the F test-retest rejects that inter-item correlations are null at statistically significant levels (*p* < 0.001 for both samples).

To minimize the role of time-allocation decisions over the life cycle (Gimenez-Nadal & Sevilla, 2012), we restrict the samples to individuals between 21 and 65 years old, and we omit individuals who filled-in their diaries on holidays, and individuals not in paid work (as we are interested only in market work time). Given that we analyze information at the episode level, we restrict the sample to time-use episodes of respondents with non-missing information on instantaneous well-being, which leaves us with a sample of 8,612 episodes from 444 women, and 6,930 episodes from 392 men in the UK, and 13,744 episodes from 9,818 women and 12,473 episodes from 9,501 men in the US. We have additionally checked for outliers using the blocked adaptive computationally efficient outlier nominators (BACON) algorithm, to detect outliers in multivariate data on the main variables (Billor et al., 2000). The BACON algorithm detects no outliers in the UK and US samples, at statistically significant levels (*p* < 0.01).

Table 1 shows the average enjoyment and episode duration for the UK, and Table 2 shows the same for the US. Among men in the UK, the average enjoyment levels during paid work, unpaid work, childcare, and leisure activities are 4.58, 4.88, 5.71, and 5.78, out of 7, respectively. For women, the equivalent enjoyment levels are 5.00, 4.77, 5.71, and 5.96. The differences between men and women are significant at standard levels in paid work episodes (p < 0.001), unpaid work (p = 0.017), and leisure (p < 0.001), with women reporting greater enjoyment while doing paid work and leisure, and lower enjoyment while doing unpaid work. Differences between women and men in terms of enjoyment during childcare are not significant. In the case of the US, the average happiness scores for men (women) in paid work, unpaid work, childcare, and leisure activities are 3.92 (3.96), 4.10 (4.11), 4.94 (4.68), and 4.56 (4.74), on a scale of 6, respectively. Differences between men and women are not significant for the happiness experienced while doing both paid and unpaid work (p = 0.335 and p = 0.788, respectively), but men seem to be happier while doing childcare than are women, and women report greater experienced happiness during leisure episodes (p < 0.001 in both cases).

**Table 1:** Summary statistics – United Kingdom

	WOMEN		MEN		DIFF.
	Mean	S. Dev.	Mean	S. Dev.	P-value
PAID WORK					
Episode duration	56.835	76.349	51.847	70.658	(0.054)
Enjoyment scale	4.999	1.223	4.584	1.453	(< 0.001)
Proportion of episodes with:					
Spouse	1.532	12.288	4.258	20.196	(< 0.001)
Children	0.666	8.138	1.496	12.143	(0.024)
Other family members	4.930	21.657	6.789	25.164	(0.025)
Non-family members	66.089	47.356	58.343	49.313	(< 0.001)
Proportion of episodes at home	7.795	26.818	11.047	31.357	(0.002)
N. periods	1,501		1,738		
UNPAID WORK					
Episode duration	20.289	19.955	22.047	25.459	(0.009)
Enjoyment scale	4.767	1.583	4.879	1.461	(0.017)
Proportion of episodes with:					
Spouse	28.651	45.221	35.740	47.938	(< 0.001)
Children	12.570	33.156	12.709	33.317	(0.891)
Other family members	17.657	38.136	15.453	36.157	(0.053)
Non-family members	9.288	29.031	7.757	26.757	(0.075)
Proportion of episodes at home	86.938	33.704	81.802	38.594	(< 0.001)
N. periods	3,446		3,191		
CHILDCARE					
Episode duration	18.964	14.523	21.754	19.571	(0.013)
Enjoyment scale	5.706	1.323	5.705	1.283	(0.992)
Proportion of episodes with:					
Spouse	33.495	47.236	54.154	49.904	(< 0.001)
Children	71.197	45.321	79.385	40.517	(0.006)
Other family members	26.861	44.359	16.615	37.279	(< 0.001)
Non-family members	11.165	31.519	4.615	21.014	(< 0.001)
Proportion of episodes at home	88.835	31.519	86.769	33.935	(0.352)
N. periods	3,047		1,676		
LEISURE					
Episode duration	30.235	31.997	36.606	40.023	(< 0.001)
Enjoyment scale	5.955	1.298	5.781	1.270	(< 0.001)
Proportion of episodes with:					
Spouse	38.218	48.599	45.534	49.808	(< 0.001)
Children	8.996	28.617	12.661	33.258	(< 0.001)
Other family members	17.092	37.650	16.233	36.881	(0.348)
Non-family members	19.037	39.265	16.797	37.390	(0.018)
Proportion of episodes at home	75.392	43.079	75.682	42.907	(0.784)
N. periods	618		325		

Note: The sample (UKTUS 2014–2015) is restricted to paid work, unpaid work, childcare, and leisure episodes of individuals between 21 and 65 years old. Time uses are measured in minutes per day. Enjoyment is measured on a 7-point scale, from 1 (“not at all”) to 7 (“very much”). T-test p-values for the differences between women and men in parentheses

**Table 2:** Summary statistics – United States

	WOMEN		MEN		DIFF.
	Mean	S. Dev.	Mean	S. Dev.	P-value
PAID WORK					
Episode duration	213.657	148.561	224.903	163.069	(0.008)
Happiness scale	3.964	1.573	3.923	1.571	(0.335)
Proportion of episodes with:					
Spouse	3.331	17.947	2.988	17.030	(0.469)
Children	3.331	17.947	1.832	13.411	(< 0.001)
Other family members	0.740	8.573	0.161	4.006	(0.001)
Non-family members	69.161	46.192	63.850	48.051	(< 0.001)
Proportion of episodes at home	16.530	37.152	16.131	36.788	(0.690)
N. periods	2,432		3,112		
UNPAID WORK					
Episode duration	51.149	64.063	54.030	70.795	(0.096)
Happiness scale	4.106	1.608	4.095	1.562	(0.788)
Proportion of episodes with:					
Spouse	18.581	38.900	27.169	44.493	(< 0.001)
Children	23.860	42.628	16.865	37.452	(< 0.001)
Other family members	3.129	17.411	2.158	14.533	(0.024)
Non-family members	8.479	27.860	8.542	27.957	(0.930)
Proportion of episodes at home	94.698	22.410	93.307	24.996	(0.022)
N. periods	4,187		2,271		
CHILDCARE					
Episode duration	35.938	49.765	44.819	60.237	(< 0.001)
Happiness scale	4.676	1.408	4.937	1.227	(< 0.001)
Proportion of episodes with:					
Spouse	19.235	39.426	39.035	48.804	(< 0.001)
Children	90.720	29.023	93.246	25.107	(0.016)
Other family members	3.881	19.319	2.105	14.362	(0.008)
Non-family members	9.168	28.865	5.877	23.530	(0.001)
Proportion of episodes at home	77.334	41.879	83.684	36.967	(< 0.001)
N. periods	1,778		1,140		
LEISURE					
Episode duration	53.103	68.181	53.937	69.566	(0.521)
Happiness scale	4.739	1.450	4.560	1.471	(< 0.001)
Proportion of episodes with:					
Spouse	30.353	45.983	34.504	47.542	(< 0.001)
Children	28.951	45.358	24.084	42.763	(< 0.001)
Other family members	5.779	23.337	4.235	20.141	(< 0.001)
Non-family members	38.208	48.594	34.723	47.613	(< 0.001)
Proportion of episodes at home	57.079	49.501	58.555	49.267	(0.113)
N. periods	5,347		5,950		

Note: The sample (ATUS SWB module 2010-2012-2013) is restricted to paid work, unpaid work, childcare, and leisure episodes of individuals between 21 and 65 years old. Time uses are measured in minutes per day. Happiness is measured on a 7-point scale, from 0 (“not at all”) to 6 (“very much”). T-test p-values for the differences between women and men in parentheses

The mean duration of episodes of paid work is about 57min for UK women, vs. 52min for UK men, with these being significant at the 90% level (p = 0.054). The average duration of unpaid work episodes is 20min for women, and 22min for men, with the difference being significant (p = 0.017). For childcare episodes, the average duration is 19min for women, and 22min for men, and the gender difference is significant at standard levels (p = 0.013). For leisure episodes, the average duration is 30 minutes for women, and 37 minutes for men, with the difference being highly significant (p < 0.001).

In the US, the average episode duration is 214 (225) minutes for women’s (men’s) paid work, 51 (54) minutes for unpaid work, 36 (45) minutes for childcare, and 53 (54) minutes for leisure. Differences between women and men in the duration of these episodes are statistically significant for the periods of paid work (p = 0.008), unpaid work (p = 0.096), and childcare (p < 0.001), but the average duration of leisure episodes is not statistically different for women and men (p = 0.521).

The UKTUS and ATUS surveys include information about who was present for all activities, distinguishing between solo activities, activities with the partner/spouse present, activities with children, activities with other family members, and activities with non-family individuals. We use this information to identify joint and solo time uses. Tables 1 and 2 show the percentage of episodes in the presence of someone else, for the 4 activities defined. In the UK, for women’s paid work episodes, 1.5% are done with the spouse, 0.7% with a child, 4.9% with other relatives, and 66.1% with others. For men, 4.3% of the episodes are done with the spouse, 1.5% with a child, 6.8% with other relatives, and 58.3% with others. In the case of unpaid work episodes of women (men), 28.7% (35.7%) are done with the spouse, 12.6% (12.7%) with a child, 17.7% (15.5%) with other relatives, and 9.3% (7.8%) with others. For childcare episodes, 33.5% (54.2%) are done with the spouse, 71.2% (79.4%) with the child, 26.9% (16.6%) with other relatives, and 11.2% (4.6%) with others. For episodes of leisure of women (men), 38.2% (45.5%) are done with the spouse, 9.0% (12.7%) with a child, 17.1% (16.2%) with other relatives, and 19.0% (16.8%) with others. For episodes of women (men) in the US, we observe that 3.3, 18.6, 19.2, and 30.4 (3.0, 27.2, 39.0 and 34.5) percent of episodes of paid work, unpaid work, childcare, and leisure is done with the spouse, respectively. Similarly, 3.3, 23.9, 90.7, and 29.0 (1.8, 16.9, 93.2, and 24.1) percent are done with a child; 0.7, 3.1, 3.9, and 5.8 (0.2, 2.2, 2.1, and 4.2) percent with other relatives, and 69.2, 8.5, 9.2, and 38.2 (63.9, 8.5, 5.9 and 34.7) percent with others. T-type test p-values for the differences between women and men in these percentages are shown in Tables 1 and 2.

Both the UKTUS and the ATUS surveys allow us to identify where the reported activities take place, differentiating among several locations (e.g., respondent’s home, workplace, restaurant, someone else’s home, stores, school, or outdoors away from home, among others). We use this information to compute a dummy variable at the episode level, which takes value 1 if activities are done at the respondent’s home, and 0 otherwise. Therefore, the variable identifies those activities done at home, versus all the activities done elsewhere. Tables 1 and 2 show the proportion of paid work, unpaid work, childcare, and leisure activities done at home, for male and female workers in the UK and the US, respectively.

In the UK, women (men) spend about 7.8 (11.0) percent of their paid work episodes at home, with the difference being statistically significant at standard levels. Conversely, women do more unpaid work at home than men, with 86.9% of women’s unpaid work episodes taking place at home, vs. 81.8% for men. The difference is statistically significant at standard levels. Regarding childcare, 88.8% of women’s episodes, and 86.8% of the men’s episodes are at home, with the gender difference being not significant at standard levels (p = 0.352). Similarly, about 75% of the leisure episodes are home-leisure, for both men and women, with a non-significant difference between them (p = 0.784). The distribution of home and not-at-home episodes in the US is different than in the UK. For instance, about 16% of the paid work episodes are at home for both men and women, with this difference not being statistically significant. Women do relatively more unpaid work at home than do men, as 94.7% of women’s unpaid work are at home, vs. 93.3% of men’s episodes, with this difference being significant (p = 0.022). Regarding childcare, 77.3 (83.7) percent of the women’s (men’s) episodes are at home, with the difference being highly significant. Finally, there are no gender differences in terms of the percent of leisure episodes done at home (57.1% for women, and 58.5% for men).

The UKTUS and ATUS allow us to examine additional control variables at the individual level, defined analogously for the UK and the US. These variables include: age, formal education, native status/being Hispanic, marital status (married or cohabiting or single), household composition (the number of family unit members, and the number of children), and employment status (identifying self-employed workers, and full-time employees). For education, we define three dummies in terms of the maximum level of formal education completed by individuals: primary, secondary, and university education. The surveys allow us to define dummies identifying geographical regions. UK regions include: “North East”, “North West & Merseyside”, “Yorkshire & Humberside”, “East midlands”, “West midlands”, “East of England”, “London”, “South East”, “South West”, “Wales”, “Scotland”, and “Northern Ireland”. US regions include: “Northeast”, “Midwest”, “South”, and “West”.

Summary statistics of the socio-demographic characteristics, and of the total time devoted to the various activities in the presence of others, are shown in Tables A1 and A2 in the Appendix. For the UK (US), men devote 230, 94, 18, and 298 (286, 52, 27, and 143) minutes to paid work, unpaid work, childcare, and leisure, respectively, while women devote 192, 139, 26, and 235 (233, 103, 39, and 136) minutes to these activities. While men devote more time to paid work (p = 0.024 in the UK, p < 0.001 in the US), women devote more time to unpaid work (p < 0.001 in both cases) and childcare (p = 0.042 and p < 0.001).^11^ Regarding the time devoted to these activities in the presence of others, we observe that most of the time spent in paid work corresponds to paid work with others (i.e., coworkers). For instance, the average time per day working with others for women in the UK (US) is about 147 (181) minutes, vs. 154 (2001) minutes among men. Differences between women and men are not significant in the UK, but highly significant (p < 0.001) in the US. For unpaid work, in the UK (US) the average time spent by women doing unpaid work is 42 (19) minutes with the spouse, 15 (26) minutes with children, 25 (3) minutes with other relatives, and 14 (8) minutes with others. Among men, the corresponding averages are 35 (16) minutes, 13 (10) minutes, 15 (1) minutes, and 8 (5) minutes.

For childcare time, women spend about 9 (9) minutes per day doing childcare with the spouse, 7 (2) minutes with other relatives, and 3 (5) minutes with others, vs. 10 (12), 2 (1) and 1 (3) minutes per day among men. In both the UK and the US, a small proportion of the childcare time (about 8 and 3min for UK women and men, and 3 and 2min for their US counterparts) is not spent in the presence of children, as some childcare activities, such as transport related to childcare, are not necessarily done with children. Finally, for leisure time in the UK (US), women spend about 91 (45) minutes per day doing leisure activities with the spouse, 19 (43) with children, 40 (7) with other relatives, and 48 (71) with non-family unit members. Among men, the corresponding minutes per day are 135 (56), 33 (38), 44 (5) and 56 (68). All these differences (p-values for the significance of such differences are shown in Tables A1 and A2 in the Appendix) may indicate that daily behaviors in terms of togetherness and the preference for joint activities are different in the US and the UK.

## Empirical Strategy

We aim to explore whether the experienced utility of workers in their daily paid work, unpaid work, childcare, and leisure activities is affected by whether such activities are done alone, or together with others (i.e., joint activities). To do so, we estimate the following linear equation using Ordinary Least Squares:

1 $${WB}_{ij}={\alpha }_{0}+{\alpha }_{1}{J}_{ij}+{\alpha }_{2}{X}_{i}+{\alpha }_{3}{E}_{ij}+{\alpha }_{4}{T}_{i}+{\epsilon }_{ij}$$

where ${WB}_{ij}$ represents the experienced utility of individual “i” while doing activity “j*”* (paid work, unpaid work, childcare, leisure). Equation(1) is estimated separately by gender, and ${J}_{ij}$ is a vector of dummy variables that identifies whether episode “j” for individual “i” is done with the (married or unmarried) partner, with children, with other household members, or with other non-household members (solo activities are considered the reference category).

${X}_{i}$ is a vector of variables controlling for the socio-demographic characteristics of individual “i” (e.g., age, education, living in couple, number of children, etc.). ${E}_{ij}$ is a vector of episode-level controls that includes the duration of the episode, where the activity took place (e.g., at home vs. other locations), if the day is a weekday (vs. weekend), and the start time of the activity. ${T}_{i}$ is the total time devoted by individual “i” to the reference activity during the diary day (paid work, unpaid work, childcare, or leisure) measured in log of minutes per day. Finally, ${\epsilon }_{ij}$ represents the error terms, and is clustered at the individual level, to take into account the heterogeneity in time-allocation decisions, as well as inter-personal differences in scales (Ferrer-i-Carbonell and Frijters, 2004).

We run several robustness checks. First, we estimate Eq.(1) using an ordered logit model. Second, we exclude from the analysis episodes between midnight and 8 am, to avoid “strange hours” (Hamermesh & Stancanelli, 2015). Third, we estimate Eq.(1) for the whole sample (i.e., men and women) and including gender scores, that is to say, a dummy variable that takes value 1 for men (0 for women), and the interactions of this dummy and the regressors of interest (the variables in ${J}_{ij}$). Fourth, we estimate Eq.(1) but including standard errors not clustered at the individual level, which allows us to compute effect magnitudes to analyze the relative variance captured by the main explanatory variables, relative to the model’s explained variance. Results indicate that the main conclusions are robust to the model, sample selection, and choice of standard errors, since the ordered logit, reduced sample, alternative gender score model, and standard errors results shown in Tables A3-A6 in the Appendix are robust to the main analysis. Furthermore, effect magnitudes are shown in Table A7 in the Appendix, indicating that model effects are relatively small. This is standard when estimating regression models with time use data, where information is subject to unobserved heterogeneity and stochastic components (Gimenez-Nadal et al., 2021).^12^

Given the number of explanatory variables included in the estimated models, we have systematically studied potential multicollinearity among explanatory variables, and among interactions of explanatory variables. In doing so, we use Variance Inflation Factors (VIF), and find overall VIF measures of 2.76 in the UK, and 3.44 in the US, when no interactions between variables are considered. When we do consider interactions, VIF measures are estimated to be 3.12 in the UK, and 4.24 in the US, both below the problematic threshold of 10, and the concern threshold of 5 (James et al., 2013). Furthermore, the only individual variables with VIF above these thresholds are education dummies in the US, as expected, given that these dummies are collinear. Thus, the estimated VIF measures suggest no potential multicollinearity issues in the empirical analysis.

## Results

Tables 3 and 4 show the main estimates of Eq.(1) in the UK and the US, respectively. Regarding paid work, we observe that in the UK, and in comparison to being alone, women report higher levels of enjoyment during paid work if it is done along with children or with non-family members, while men report lower levels of enjoyment when paid work is done with children. In the case of the US, we find no happiness differences for women when paid work is done with others, in comparison with being alone, while men report higher levels of happiness during paid work when it is done with other family members. When challenged by lockdowns, such as the one caused by COVID-19, implying an increase in teleworking for many workers, and thus more time in paid work in company with the spouse/partner and the children, and less time in the presence of others (both family and non-family members), men in both the UK and the US would reduce their experienced utility during paid work time, while women would increase theirs. It is also interesting that women in the UK report higher levels of experienced utility when paid work is done in the presence of children, which may be a consequence of the “double burden”, or second shift, that they face - especially for those women who work full-time and have children (Gimenez-Nadal & Sevilla, 2011). If those women are able to take care of their children while they are working - which would obviously be the case in a ‘lockdown’ situation - the difficulties in balancing work and household responsibilities would be reduced, which would clearly improve women’s well-being. Furthermore, we find that working at home is not correlated with worker experienced enjoyment in a significant way in the UK, nor among US men. Estimates show that episodes of paid work at home for women workers are correlated with lower happiness, relative to episodes of paid work not at home, which may indicate that women may face higher risks of work interruptions during lockdowns, given the presence of children at home. This last finding is consistent with prior evidence showing that women get more frequent interruptions while teleworking (Adams-Prassl, 2020).

**Table 3:** Estimates for the United Kingdom

VARIABLES	PAID WORK		UNPAID WORK		CHILDCARE		LEISURE	
	**Women**	**Men**	**Women**	**Men**	**Women**	**Men**	**Women**	**Men**
	**(1)**	**(2)**	**(3)**	**(4)**	**(5)**	**(6)**	**(7)**	**(8)**
**WITH**:								
With the spouse	-0.730	0.211	0.346**	0.102	-0.176	-0.048	0.291**	0.290***
	(0.463)	(0.418)	(0.133)	(0.142)	(0.161)	(0.217)	(0.091)	(0.085)
	[0.117]	[0.613]	[0.010]	[0.472]	[0.275]	[0.825]	[0.001]	[0.001]
With children	0.994**	-3.457***	0.152	0.111	-	-	0.107	0.214
	(0.319)	(0.730)	(0.171)	(0.206)			(0.121)	(0.156)
	[0.002]	[0.000]	[0.377]	[0.591]			[0.380]	[0.171]
With others (family unit)	-0.149	0.230	0.419**	-0.044	0.155	0.150	0.038	-0.010
	(0.244)	(0.438)	(0.128)	(0.157)	(0.216)	(0.282)	(0.148)	(0.108)
	[0.541]	[0.600]	[0.001]	[0.779]	[0.475]	[0.595]	[0.799]	[0.925]
With others (non-fam. unit)	0.358*	0.029	0.619***	0.389*	0.470*	0.490	0.217*	0.296**
	(0.142)	(0.158)	(0.144)	(0.185)	(0.196)	(0.356)	(0.091)	(0.095)
	[0.012]	[0.857]	[0.000]	[0.036]	[0.018]	[0.173]	[0.018]	[0.002]
**EPISODE CONTROLS**:								
Where: at home	-0.182	0.066	-0.254	0.030	0.545	0.163	-0.268**	-0.158
	(0.288)	(0.322)	(0.146)	(0.134)	(0.313)	(0.307)	(0.089)	(0.089)
	[0.528]	[0.837]	[0.083]	[0.823]	[0.084]	[0.596]	[0.003]	[0.078]
Start time 0 = 4am	0.001*	0.000	0.000**	0.000*	0.001	0.001**	0.000***	0.000**
	(0.000)	(0.000)	(0.000)	(0.000)	(0.000)	(0.000)	(0.000)	(0.000)
	[0.018]	[0.499]	[0.002]	[0.022]	[0.064]	[0.005]	[0.001]	[0.001]
Episode duration	-0.002**	-0.002**	-0.001	-0.001	0.015**	0.006	0.002	0.003***
	(0.001)	(0.001)	(0.002)	(0.002)	(0.005)	(0.003)	(0.001)	(0.001)
	[0.006]	[0.006]	[0.488]	[0.518]	[0.002]	[0.068]	[0.084]	[0.000]
All other controls	Yes	Yes	Yes	Yes	Yes	Yes	Yes	Yes
Observations	1,501	1,738	3,047	1,676	618	325	3,446	3,191
R-squared	0.099	0.117	0.090	0.056	0.207	0.288	0.065	0.081
Adjusted R-squared	0.087	0.106	0.084	0.044	0.182	0.241	0.060	0.075
F test *p*-value	< 0.001	< 0.001	< 0.001	< 0.001	< 0.001	< 0.001	< 0.001	< 0.001
AIC	4919.2	6037.9	11196.9	5865.5	2049.7	1019.8	11741.2	10423.6
BIC	5030.8	6158.0	11329.4	5984.8	2138.2	1099.3	11876.4	10557.1

Note: The sample (UKTUS 2014–2015) is restricted to paid work, unpaid work, childcare, and leisure episodes of individuals between 21 and 65 years old. The dependent variable is the subjective enjoyment of episodes, which takes values from 1 (“not at all”) to 7 (“very much”). Robust standard errors, clustered at the individual level, in parentheses. T-test p-values in brackets. Additional coefficients shown in Table A8 in the Appendix. *** Significant at the 99.9% level; ** significant at the 99% level; * significant at the 95% level

**Table 4:** Estimates for the United States

VARIABLES	PAID WORK		UNPAID WORK		CHILDCARE		LEISURE	
	**Women**	**Men**	**Women**	**Men**	**Women**	**Men**	**Women**	**Men**
	**(1)**	**(2)**	**(3)**	**(4)**	**(5)**	**(6)**	**(7)**	**(8)**
**WITH**:								
With the spouse	0.195	-0.417	0.267*	0.422***	0.184	0.324**	0.113	0.378***
	(0.251)	(0.447)	(0.108)	(0.123)	(0.112)	(0.099)	(0.114)	(0.083)
	[0.436]	[0.351]	[0.013]	[0.001]	[0.101]	[0.001]	[0.322]	[0.000]
With children	0.360	-0.006	0.338**	0.048	-	-	0.373***	0.361***
	(0.193)	(0.228)	(0.113)	(0.144)			(0.080)	(0.076)
	[0.062]	[0.978]	[0.003]	[0.738]			[0.000]	[0.000]
With others (family unit)	0.615	1.748***	0.168	0.120	0.555**	-0.541	0.333	0.277
	(0.454)	(0.319)	(0.218)	(0.352)	(0.181)	(0.451)	(0.179)	(0.163)
	[0.176]	[0.000]	[0.441]	[0.733]	[0.002]	[0.231]	[0.063]	[0.088]
With others (non-fam. unit)	0.144	0.102	0.553***	0.511*	0.258	0.252	0.343***	0.170
	(0.111)	(0.106)	(0.133)	(0.201)	(0.189)	(0.219)	(0.086)	(0.091)
	[0.194]	[0.337]	[0.000]	[0.011]	[0.172]	[0.249]	[0.000]	[0.062]
**EPISODE CONTROLS**:								
Where: at home	-0.464**	0.185	-0.005	0.127	-0.041	0.167	-0.051	-0.101
	(0.157)	(0.148)	(0.287)	(0.201)	(0.140)	(0.185)	(0.076)	(0.086)
	[0.003]	[0.211]	[0.986]	[0.528]	[0.769]	[0.368]	[0.498]	[0.243]
Start time 0 = 4am	-0.001***	-0.000	-0.000***	-0.000	0.000	-0.000	0.000	0.000
	(0.000)	(0.000)	(0.000)	(0.000)	(0.000)	(0.000)	(0.000)	(0.000)
	[0.000]	[0.102]	[0.001]	[0.533]	[0.069]	[0.836]	[0.186]	[0.054]
Episode duration	-0.001	-0.001	0.001	-0.001	0.001**	0.000	0.000	0.000
	(0.000)	(0.000)	(0.001)	(0.001)	(0.000)	(0.001)	(0.000)	(0.000)
	[0.070]	[0.074]	[0.385]	[0.263]	[0.002]	[0.413]	[0.476]	[0.323]
All other controls	Yes	Yes	Yes	Yes	Yes	Yes	Yes	Yes
Observations	2,432	3,112	4,187	2,271	1,778	1,140	5,347	5,950
R-squared	0.086	0.029	0.056	0.076	0.083	0.084	0.063	0.067
Adjusted R-squared	0.078	0.022	0.052	0.068	0.073	0.068	0.060	0.064
F test *p*-value	< 0.001	< 0.001	< 0.001	< 0.001	< 0.001	< 0.001	< 0.001	< 0.001
AIC	8785.3	11528.1	15643.2	8300.4	6049.7	3551.1	18355.4	20644.3
BIC	8912.8	11661.0	15782.7	8426.4	6164.8	3656.9	18500.3	20791.5

Note: The sample (ATUS SWB module 2010-2012-2013) is restricted to paid work, unpaid work, childcare, and leisure episodes of individuals between 21 and 65 years old. The dependent variable is the happiness level of episodes, which takes values from 0 (“not at all”) to 6 (“very much”). Robust standard errors, clustered at the individual level, in parentheses. T-test p-values in brackets. Additional coefficients shown in Table A9 in the Appendix. *** Significant at the 99.9% level; ** significant at the 99% level; * significant at the 95% level

Regarding the time devoted to unpaid work, women in the UK and the US report higher enjoyment/happiness during these activities if they are done with the spouse, or in the presence of children or non-family members, in comparison to being alone. The consequences of confinement on the experienced utility of women while doing unpaid work, with more time with the spouse and children and less time with non-family members, is a priori undetermined. Men in the UK report higher levels of enjoyment during unpaid work when the activity is done with non-family members, while in the US men report higher levels of happiness when the activity is done with the spouse/partner, or with non-family members, in comparison with doing the activity alone. Regarding the location of housework activities, we observe a non-statistically significant correlation between doing unpaid work at home and experienced enjoyment or happiness of workers. These results imply that during the confinement of lockdown, men in the UK obtain lower experienced utility than in normal circumstances, given that they spend less time with non-family members. In the case of men in the US, the final effect is undetermined.

In the case of childcare time, women in the UK report higher enjoyment when the activity is done with non-family members, while women in the US report higher levels of happiness when the activity is done with other family members, in comparison to being alone. Given the decrease in the time spent in childcare with other family members, and with non-family members, women have lower experienced utility during the lockdown. In the case of men in the UK, we find no differences between the time devoted to childcare with others or alone, while men in the US report higher levels of happiness when the childcare is done with the spouse. The latter finding indicates that men are better off in terms of experienced utility during childcare activities, given the increase in the presence of the spouse during childcare activities. Furthermore, there are no differences in the experienced enjoyment depending on whether childcare activities are done at home or elsewhere, as the associated coefficients are not statistically significant at standard levels.

Finally, regarding the time devoted to leisure, we find that both men and women in the UK report higher levels of enjoyment when the activity is done with the spouse, or in the presence of non-family members. Conversely, in the US men, but not women, report higher levels of happiness if leisure activities are done in the presence of the spouse, whereas women, but not men, reporter higher levels of happiness if the activity is done in the presence of non-family members. Both men and women in the US also report increased happiness if leisure activities are done with children. Given that leisure time with family members increases during the Covid-19 lockdown, but leisure time with non-family members decreases, the consequences for the experienced utility of individuals are undetermined. Furthermore, women in the UK report enjoying their leisure episodes less when they took place at home, therefore contributing to the undetermined result. In the US, the coefficients identifying episodes at home are not significant for women or for men.

It is important to note that the estimated models have low ${R}^{2}$ statistics, indicating that they explain relatively low rates of variance. However, this is standard when analyzing data from Time Use Surveys (Ferrer-i-Carbonell and Frijters, 2004) ; Gimenez-Nadal et al., 2021). Furthermore, we have analyzed which models should be preferred among the baseline estimates and the set of proposed robustness checks. Using the ${R}^{2}$, and the adjusted-${R}^{2}$, estimates suggest that all models perform similarly, with slight changes in these statistics. We similarly estimated information criteria measures to study competition across models, and computed the AIC and BIC measures. These statistics suggest that ordered logit models slightly outperform OLS estimates, although we rely on the baseline OLS estimates, for the sake of simplicity of interpretation, and given that conclusions are robust. On the other hand, AIC and BIC measures also suggest that the estimates in which both men and women are pooled in the same sample are relatively worse than the baseline estimates, and that estimates on the reduced sample are slightly preferable. Nevertheless, as these results are based on different samples, the comparisons should be considered cautiously.

## A Lockdown Simulation

Hamermesh (2020) simulates a lockdown and estimates its impact on life satisfaction, using data from the ATUS. The simulation is based on specific changes in time use of specific respondents, namely married individuals with no children, and singles aged 30 or older with no children. The author redefines all the “with whom” variables for married individuals to include the partner, and the same variable to time alone for singles. He also assumes that one third of the paid work time is lost, that such time is assigned to leisure activities, and that income is also cut by one third. He then computes OLS estimates under these changes, and predicts a simulated life satisfaction ladder (i.e., a subjective measure of cognitive well-being), which is predicted imposing the latter changes. Hamermesh (2020) then compares the predicted life satisfaction ladder under the simulated lockdown conditions, and the reported life satisfaction ladder. These assumptions translate into an increase in married couples’ general life satisfaction, but a decrease in that of singles.

In our analysis, we follow a comparable simulation. However, as Hamermesh (2020) carries out the analysis on a life satisfaction ladder, and not on affective measures of experienced utility, some differences emerge. First, we estimate Eq.(1) on real data, from where we get the effect of the presence of others, and/or being at home, on the experienced wellbeing during different activities (paid work, unpaid work, childcare, and leisure). These are the results shown in Tables 3 and 4, for the UK and the US, respectively. We then simulate a strict lockdown, and in doing so we impose several changes on respondents’ diaries.

First, we assume that all the time categories remain unchanged. That is to say, all the paid work activity episodes remain as paid work episodes, all the unpaid work episodes remain as unpaid work, all the childcare episodes remain as childcare, and all the leisure episodes remain as leisure. However, we do make changes in the variables reflecting with whom the activities are done (alone, with partners, with children, with other relatives, with others), and in the location of episodes, for all the episodes of the samples. All the episodes are computed as if they took place at the respondent’s home, generating a simulated variable for episode location indicating that all episodes take place at homeFor married individuals without children, and not cohabiting with others, all the episodes are redefined as “with the partner”. For married individuals without children, but cohabiting with others, all the episodes are redefined as “with the partner” and “with others (family unit)”. For married individuals with children, and not cohabiting with others, all the episodes are redefined as “with the partner” and “with children”. For married individuals with children, and cohabiting with others, all the episodes are redefined as “with the partner”, “with children”, and “with others (family unit)”. For singles who live alone, all episodes are redefined as “alone”. For singles who live with children, and not cohabiting with others, all episodes are redefined as “with children”. For singles without children, but cohabiting with other relatives, all episodes are recoded as “with others (family unit)”. Finally, for single individuals with children and who cohabit with relatives other than the partner and children, all the episodes are redefined as “with children”, and “with other relatives”.

Once the synthetic sample of episodes is created, we predict the enjoyment/happiness arising from these episodes, but using the coefficients that were initially obtained from Eq.(1) regarding who else is present, being at home, and socio-demographic characteristics.^13^ Table 5 shows the average reported enjoyment/happiness of respondents in their daily activities (i.e., the real episodes), the average computed enjoyment/happiness under the simulated lockdown (i.e., the synthetic episodes), along with the differences in average enjoyment/happiness during the time devoted to paid work, unpaid work, childcare, and leisure for the synthetic episodes, and for the real episodes. Positive values indicate increases in experienced enjoyment/happiness induced by the simulated lockdown, while negative values indicate decreases. We also report t-type test p-values for the statistical significance of the estimated differences.

**Table 5:** Lockdown simulation

		PAID WORK		UNPAID WORK		CHILDCARE		LEISURE	
		**Women**	**Men**	**Women**	**Men**	**Women**	**Men**	**Women**	**Men**
		**(1)**	**(2)**	**(3)**	**(4)**	**(5)**	**(6)**	**(7)**	**(8)**
**UNITED KINGDOM**									
Married/cohabiting									
With children	Reported	4.691	4.517	4.478	4.732	5.706	5.705	5.867	5.724
	Simulated	4.621	1.546	4.738	4.874	5.551	5.695	6.003	5.857
	Difference	-0.070	-2.971***	0.260***	0.142*	-0.151**	-0.011	0.136***	0.133***
	*p*-value	(0.268)	(< 0.001)	(< 0.001)	(0.013)	(0.006)	(0.873)	(< 0.001)	(< 0.001)
Without children	Reported	5.140	4.401	4.942	4.912	-	-	5.914	5.718
	Simulated	3.790	4.739	5.053	4.957	-	-	5.896	5.770
	Difference	-1.350***	0.338***	0.111*	0.044	-	-	-0.019	0.052
	*p*-value	(< 0.001)	(< 0.001)	(0.021)	(0.446)			(0.645)	(0.161)
Singles									
With children	Reported	5.145	5.446	4.716	4.989	5.659	6.500	6.044	5.976
	Simulated	5.514	1.887	4.876	5.003	5.754	6.178	6.101	5.900
	Difference	0.370***	-3.559***	0.161	0.015	0.095	-0.321	0.057	-0.077
	*p*-value	(< 0.001)	(< 0.001)	(0.053)	(0.925)	(0.459)	(0.519)	(0.289)	(0.345)
Without children	Reported	5.084	4.826	5.114	5.068	-	-	6.083	5.968
	Simulated	4.599	4.998	5.125	5.059	-	-	6.023	5.846
	Difference	-0.485***	0.172*	0.011	-0.014	-	-	-0.060	-0.122**
	*p*-value	(< 0.001)	(0.042)	(0.875)	(0.856)			(0.141)	(0.006)
**UNITED STATES**									
Married/cohabiting									
With children	Reported	4.032	3.897	4.114	4.199	4.651	4.930	4.856	4.680
	Simulated	4.290	3.956	4.492	4.461	4.897	5.228	5.076	5.121
	Difference	0.258***	0.059	0.378***	0.262***	0.246***	0.297***	0.221***	0.441***
	*p*-value	(< 0.001)	(0.205)	(< 0.001)	(< 0.001)	(< 0.001)	(< 0.001)	(< 0.001)	(< 0.001)
Without children	Reported	4.022	4.097	4.301	4.150	-	-	4.812	4.622
	Simulated	3.837	4.192	4.363	4.402	-	-	4.819	4.862
	Difference	-0.185*	0.095	0.061	0.252**	-	-	0.007	0.240***
	*p*-value	(0.014)	(0.208)	(0.278)	(0.003)			(0.880)	(< 0.001)
Singles									
With children	Reported	4.074	3.788	4.089	4.192	4.737	4.984	4.708	4.615
	Simulated	4.194	5.297	4.351	4.219	5.017	4.801	5.095	4.932
	Difference	0.120	1.509***	0.262***	0.027	0.281***	-0.183	0.387***	0.308***
	*p*-value	(0.106)	(< 0.001)	(< 0.001)	(0.795)	(< 0.001)	(0.119)	(< 0.001)	(< 0.001)
Without children	Reported	3.802	3.890	3.949	3.918	-	-	4.595	4.359
	Simulated	3.541	4.685	4.107	3.982	-	-	4.725	4.479
	Difference	-0.262***	0.795***	0.158**	0.064	-	-	0.130***	0.120***
	*p*-value	(< 0.001)	(< 0.001)	(0.004)	(0.268)			(< 0.001)	(0.001)

Note: The samples (UKTUS 2014–2015; ATUS SWB module 2010-2012-2013) are restricted to paid work, unpaid work, childcare, and leisure episodes of individuals between 21 and 65 years old. Differences computed between the experienced enjoyment/happiness predicted when simulating a lockdown, minus the experienced enjoyment/happiness reported by respondents. Positive values indicate estimated increases in enjoyment while in a simulated lockdown. Lockdown simulation includes: (1) For individuals cohabiting, all the time is assigned to: “with the spouse”. (2) For single individuals, all the time is assigned as: “alone”. (3) For individuals with children, all the time is assigned to: “with children”. (4) All the time is assigned to: “where: at home”. (5) For working individuals, we compute a cut in paid work of 1/3 of the time, which is assigned to leisure (Hamermesh, 2020). T-test p-values in brackets. *** Significant at the 99.9% level; ** significant at the 99% level; * significant at the 95% level

The results for the UK (Panel A) show that, for married individuals with children, the simulated lockdown has a negative impact on the instant enjoyment associated with paid work activities, though it is only statistically significant among men. On the other hand, the lockdown has a positive impact on the instant enjoyment associated with unpaid work, for both women and men. Childcare (leisure) episodes of married women, however, are less (more) enjoyable. For married individuals with children, the lockdown has an opposite effect on women and men, as the former seem to enjoy less their paid work episodes, while the latter enjoy them more. Among single workers with children, the simulation shows that a lockdown has a positive impact on women’s enjoyment during paid work episodes, but a negative impact on that of men. The results for single workers without children are the opposite. Finally, the leisure episodes of single men without children seem to be less enjoyable during the simulated lockdown.

Panel B shows the results for the US. The simulated lockdown has a statistically significant impact on married women’s experienced happiness while doing paid work, but not on married men. However, this impact depends on the presence of children, as it is positive for married women with children, but negative for married women without children. The simulated confinement also has a positive impact on the level of happiness while doing unpaid work and childcare activities of married women and men with children, and on unpaid work episodes of married men (but not women) without children. Leisure episodes also seem to be more enjoyable for couples with children, and for married men without children. For singles, the confinement has a positive impact on single men’s happiness while doing paid work, but a negative impact on single women without children. On the other hand, single women’s happiness while doing unpaid work increases during the simulated lockdown, while that of men seems to be unaffected. Single women with children also enjoy more their childcare episodes during the simulation, while the happiness while doing leisure increases for both single women and men, with and without children.

Table 5 shows that the simulated lockdown has a heterogeneous impact on individual experienced utility, which depends not only on the respondents’ gender and household composition, but also on the country analyzed. Overall, taking together all the episodes and activities, the reported enjoyment of women (men) in the UK was 5.33 (5.26) out of 7, and the reported happiness in the US was 4.38 (4.29), out of 6. The simulated lockdown leads to predicted enjoyment of 5.27 for women and 4.96 for men in the UK, and predicted happiness values of 4.56 for US women and 4.69 for US men. These figures indicate a decrease in experienced utility in the UK, and an increase in the experienced utility of US individuals (with the differences being statistically significant at standard levels).

These heterogeneous affects are interesting, across both genders and countries. We note that in the UK men with children would have lower levels of enjoyment in comparison to a normal situation, while those without children would report higher levels. This may indicate that fathers may have needed to increase their care responsibilities under lockdown and school closures (Sevilla & Smith, 2020) and thus their paid work activities may have been more intertwined with childcare responsibilities (more interruptions), leading to lower levels of well-being. But, in the absence of children, telework would have allowed them to be better off than in normal circumstances (Gimenez-Nadal et al., 2021). On the other hand, women in the UK would report lower levels of enjoyment during paid work if they did not have children, which may be a consequence of the higher proportion of women working in essential occupations and thus having to go to their workplaces (explaining the increased childcare time of fathers).

But in the case of the US, single men with and without children would report higher levels of enjoyment while working during lockdowns, which contrasts with the results in the UK. This may indicate that this group may prefer isolation to social contact in a normal situation. Furthermore, women without children would report lower levels of enjoyment, while married women with children would report higher levels of enjoyment while working, in comparison to a normal situation. This may indicate that women may be taking most responsibilities for childcare during lockdowns and school closures, and thus working from home may allow them to better balance their work and household responsibilities.

Such cross-country differences in the results of the simulation may be a reflection that both countries have different cultures or roles regarding who cares for the children. For instance, Fisher and Robinson (2011) compare the time devoted to different activities, by individuals between the ages of 18 and 64 in twenty three countries, including the UK and the US, and observe that the gender gap in childcare time (i.e., women devote more time than men) is one hour more for the US, which may indicate that gender roles regarding childcare time are more traditional in the US. Other factors explaining cross-country differences could be related to the occupational composition of the countries, since if one of the countries has a higher proportion of jobs that can be done via telework, the negative consequences of lockdown would be different. Other factors could be used to explain differences in cross-country in lockdowns (e.g., what occupations are considered essential, duration of lockdowns, restrictions applied to the population). Despite that we offer three factors that could be explored in order to explain cross-country differences in the results of simulations, there are many others that we omit. Future research should focus on exploring these different factors.

## Conclusion

This paper analyzes the potential differences in the experienced wellbeing of women and men in their daily activities, with a focus on the presence of others and being at home, while doing paid work, unpaid work, childcare, and leisure. We use time use diary data from the UKTUS for the years 2014–2015, and the ATUS SWB module for the years 2010, 2012, and 2013, which provide information on the experienced utility associated with a range of episodes. Our results reveal gender differences in the experienced utility of individuals during paid work, unpaid work, childcare, and leisure activities. Furthermore, our simulation exercise shows that, in a lockdown situation, the experienced wellbeing of women may increase compared to that of men, given that more time is spent with the spouse/partner and children and less time with other family members and non-family members. This suggests that confinements, such as those generated by the COVID-19 pandemic, may have a differential impact on men and women, as men seem to be less sensitive to whether their daily activities are done alone, or not.

We report cross-country differences in these relationships, as estimated differences between joint and solo activities differ for men and women in the UK and in the US. Experienced utility in the UK seems to be more sensitive to ‘togetherness’ than in the US, suggesting the existence of heterogeneity between countries, as both men and women appear to enjoy the presence of their partner in certain ways, especially while engaged in leisure activities in the UK, while results in the US do not support this conclusion. Hence, the impact of confinement on daily behaviors may affect women and men differentially, depending on the context and the country analyzed. Our simulation exercise supports this argument, since it reveals asymmetric effects on individual experienced utility for the US and the UK, as the lockdown has a different impact in those countries, in addition to different effects for women and men. This suggests that the same measures aimed at improving individual welfare during a pandemic may have different effects in different economies, countries, and regions, and the measures policy-makers contemplate should be studied individually.

Our conclusions must be taken with caution, given that we are assuming that the time devoted to these four activities does not change with confinement. The relative gains in experienced utility of women, arising from spending more time with family members, may be countered by the fact that the COVID-19 outbreak has exacerbated the need for care work within the home, due to school closures and to the significant numbers of people contracting the virus and requiring care at home. The gender asymmetry, favoring women, in the gains in utility from more time spent with family members may compensate for the negative consequences of the extra workload for women in these difficult times. This limitation is closely related to the fact that we do not have reliable time use data on a confinement situation, including affective subjective well-being information. We need information not collected during a lockdown, and then results should be interpreted cautiously. It is important to note that the ATUS has recently launched the 2020 wave of the survey, including information on time use during the period between mid-May and the 31st December; however, this data does not contain information on subjective well-being, and given the impact of the COVID-19 on the sampling method, annual estimates and comparisons are not recommended.^14^

Other limitations include that the data is cross-sectional and, as a consequence, the results cannot be interpreted as causal; we can only report conditional correlations. Furthermore, the analysis focuses on “daily” experienced utility, which is a measure of instantaneous well-being. As such, the consequences are not applicable to the long run and will likely vanish as the lockdown is lifted (Hamermesh, 2020). However, it would be interesting to analyze whether these differences in experienced utility have any long-term consequences in, for example, an analysis of stress, depression, or mental health problems after the lockdown. Finally, the analysis is restricted to the UK and the US because of data availability, and thus results cannot be generalized to the general population in other economies, especially those not in the Western, Educated, Industrialized, Rich and Democratic (WEIRD) world.

.

## Appendix

**Table A1:** Additional summary statistics – United Kingdom

	WOMEN		MEN		DIFF.
	Mean	S. Dev.	Mean	S. Dev.	P-value
TIME USE VARIABLES					
Paid work time	192.140	230.774	229.872	251.244	(0.024)
Paid work with:					
Spouse	2.387	22.166	7.577	42.178	(0.024)
Children	0.676	13.770	2.066	26.347	(0.331)
Other family members	12.590	64.278	12.245	68.898	(0.940)
Non-family members	146.734	210.845	154.235	217.051	(0.613)
Unpaid work time	139.234	113.499	94.260	94.072	(< 0.001)
Unpaid work with:					
Spouse	41.734	71.121	35.102	61.855	(0.153)
Children	15.315	47.839	12.602	41.498	(0.384)
Other family members	24.685	60.290	14.668	43.178	(0.007)
Non-family members	14.257	40.999	7.959	29.780	(0.012)
Childcare time	26.396	66.132	18.036	50.012	(0.042)
Childcare with:					
Spouse	8.559	27.902	10.077	29.880	(0.448)
Children	17.950	55.620	15.128	46.685	(0.430)
Other family members	7.095	25.495	2.296	12.401	(0.000)
Non-family members	3.288	17.209	0.765	6.742	(0.007)
Leisure time	234.662	151.649	297.985	185.762	(< 0.001)
Leisure with:					
Spouse	91.622	124.064	135.408	148.939	(< 0.001)
Children	19.414	55.984	32.730	88.271	(0.009)
Other family members	40.428	75.578	44.158	98.590	(0.537)
Non-family members	48.423	95.467	55.536	103.853	(0.303)
SOCIO-DEMOGRAPHICS					
Age	39.777	12.239	41.916	12.503	(0.013)
Educ.: Primary	0.018	0.133	0.043	0.204	(0.032)
Educ.: Secondary	0.345	0.476	0.306	0.461	(0.237)
Educ.: University	0.637	0.481	0.651	0.477	(0.693)
Being native	0.919	0.273	0.921	0.270	(0.916)
Living in couple	0.628	0.484	0.747	0.435	(< 0.001)
Family size	2.899	1.272	3.013	1.288	(0.199)
Number of children	0.718	0.936	0.740	0.977	(0.748)
Full time worker	0.671	0.470	0.893	0.310	(< 0.001)
Self-employed worker	0.007	0.082	0.023	0.150	(0.049)
N. individuals	444		392		

Note: The sample (UKTUS 2014–2015) is restricted to individuals with episodes of paid work, unpaid work, childcare, and leisure, between 21 and 65 years old. Time uses are measured in minutes per day

**Table A2:** Additional summary statistics – United States

	WOMEN		MEN		DIFF.
	Mean	S. Dev.	Mean	S. Dev.	P-value
TIME USE VARIABLES					
Paid work time	233.451	249.346	285.751	273.925	(< 0.001)
Paid work with:					
Spouse	4.638	36.577	4.878	39.757	(0.662)
Children	4.579	38.140	2.919	30.943	(< 0.001)
Other family members	0.695	16.721	0.658	16.825	(0.878)
Non-family members	180.890	236.983	200.943	257.894	(< 0.001)
Unpaid work time	103.350	119.857	52.464	88.297	(< 0.001)
Unpaid work with:					
Spouse	19.036	53.733	15.596	48.671	(< 0.001)
Children	25.802	64.845	10.080	36.835	(< 0.001)
Other family members	2.973	21.995	1.164	12.580	(< 0.001)
Non-family members	8.317	35.294	5.056	27.640	(< 0.001)
Childcare time	39.052	81.304	26.977	69.843	(< 0.001)
Childcare with:					
Spouse	9.428	37.573	11.584	42.722	(0.002)
Children	35.631	78.359	25.027	67.166	(< 0.001)
Other family members	1.526	15.058	0.705	12.060	(< 0.001)
Non-family members	4.666	26.348	2.515	20.119	(< 0.001)
Leisure time	135.991	124.863	143.252	132.572	(< 0.001)
Leisure with:					
Spouse	45.406	88.687	56.478	96.607	(< 0.001)
Children	43.193	83.772	38.344	80.154	(< 0.001)
Other family members	7.412	38.501	5.397	32.902	(< 0.001)
Non-family members	71.328	114.510	67.524	116.094	(0.022)
SOCIO-DEMOGRAPHICS					
Age	41.811	12.031	41.862	11.770	(0.762)
Educ.: Primary	0.011	0.105	0.017	0.129	(< 0.001)
Educ.: Secondary	0.417	0.493	0.400	0.490	(0.098)
Educ.: University	0.572	0.495	0.584	0.493	(0.013)
Being Hispanic	0.848	0.359	0.819	0.385	(< 0.001)
Living in couple	0.508	0.500	0.590	0.492	(< 0.001)
Family size	2.936	1.470	3.027	1.557	(< 0.001)
Number of children	1.006	1.117	0.986	1.170	(0.246)
Full time worker	0.733	0.442	0.887	0.317	(< 0.001)
Self-employed worker	0.077	0.267	0.114	0.318	(< 0.001)
N. individuals	9,818		9,501		

Note: The sample (ATUS SWB module 2010-2012-2013) is restricted to individuals with episodes of paid work, unpaid work, childcare, and leisure, between 21 and 65 years old. Time uses are measured in minutes per day

**Table A3:** Ordered logit estimates

	PAID WORK		UNPAID WORK		CHILDCARE		LEISURE	
	**Women**	**Men**	**Women**	**Men**	**Women**	**Men**	**Women**	**Men**
	**(1)**	**(2)**	**(3)**	**(4)**	**(5)**	**(6)**	**(7)**	**(8)**
**UNITED KINGDOM**								
With the spouse	-0.843	0.179	0.460**	0.174	-0.320	-0.271	0.466**	0.520***
	(0.762)	(0.711)	(0.157)	(0.198)	(0.256)	(0.449)	(0.154)	(0.145)
	[0.269]	[0.802]	[0.003]	[0.382]	[0.211]	[0.546]	[0.003]	[0.000]
With children	1.263*	-4.818***	0.143	0.161	-	-	0.141	0.352
	(0.495)	(1.447)	(0.214)	(0.273)			(0.205)	(0.251)
	[0.011]	[0.001]	[0.505]	[0.555]			[0.491]	[0.161]
With others (family unit)	-0.127	0.224	0.543***	-0.096	0.174	0.294	0.285	-0.002
	(0.319)	(0.764)	(0.155)	(0.212)	(0.330)	(0.449)	(0.193)	(0.180)
	[0.690]	[0.769]	[0.000]	[0.649]	[0.598]	[0.512]	[0.139]	[0.991]
With others (non-fam. unit)	0.518*	0.057	0.768***	0.614*	0.627	0.761	0.394*	0.557**
	(0.210)	(0.221)	(0.184)	(0.260)	(0.356)	(0.720)	(0.184)	(0.175)
	[0.013]	[0.797]	[0.000]	[0.018]	[0.078]	[0.291]	[0.033]	[0.001]
All other controls	Yes	Yes	Yes	Yes	Yes	Yes	Yes	Yes
Observations	1,501	1,738	3,047	1,676	618	325	3,446	3,191
R-squared	-	-	-	-	-	-	-	-
Adjusted R-squared	-	-	-	-	-	-	-	-
F test *p*-value	-	-	-	-	-	-	-	-
AIC	4785.2	5908.7	10658.1	5670.7	1793.0	908.5	9228.6	9080.8
BIC	4923.3	6056.1	10826.7	5817.1	1903.6	1006.9	9400.7	9250.7
**UNITED STATES**								
With the spouse	0.267	-0.461	0.302*	0.511***	0.281	0.512**	0.172	0.537***
	(0.374)	(0.503)	(0.134)	(0.152)	(0.169)	(0.179)	(0.139)	(0.122)
	[0.475]	[0.359]	[0.024]	[0.001]	[0.096]	[0.004]	[0.218]	[0.000]
With children	0.522	-0.117	0.404**	0.092	-	-	0.461***	0.527***
	(0.277)	(0.270)	(0.137)	(0.175)			(0.115)	(0.123)
	[0.060]	[0.665]	[0.003]	[0.599]			[0.000]	[0.000]
With others (family unit)	1.134	3.503**	0.155	0.342	0.483	-0.951	0.518*	0.423
	(0.910)	(1.223)	(0.257)	(0.404)	(0.336)	(0.784)	(0.261)	(0.233)
	[0.213]	[0.004]	[0.547]	[0.397]	[0.151]	[0.225]	[0.047]	[0.069]
With others (non-fam. unit)	0.183	0.068	0.561***	0.655*	0.484	0.510	0.540***	0.272*
	(0.130)	(0.123)	(0.158)	(0.260)	(0.255)	(0.342)	(0.117)	(0.126)
	[0.158]	[0.577]	[0.000]	[0.012]	[0.058]	[0.136]	[0.000]	[0.031]
All other controls	Yes	Yes	Yes	Yes	Yes	Yes	Yes	Yes
Observations	2,432	3,112	4,187	2,271	1,778	1,140	5,347	5,950
R-squared	-	-	-	-	-	-	-	-
Adjusted R-squared	-	-	-	-	-	-	-	-
F test *p*-value	-	-	-	-	-	-	-	-
AIC	8266.3	10899.7	14193.7	7678.4	5099.7	2842.9	14895.5	17748.9
BIC	8422.8	11062.9	14364.9	7833.1	5242.2	2973.9	15073.3	17929.5

Note: The samples (UKTUS 2014–2015; ATUS SWB module 2010-2012-2013) are restricted to market work, leisure, housework, and childcare episodes of individuals between 21 and 65 years old. The dependent variable is the subjective enjoyment of episodes, which takes values from 1 (“not at all”) to 7 (“very much”), or the affective results of episodes, which take values from 0 (“not at all”) to 6 (“very much”). Robust standard errors, clustered at the individual level, in parentheses. T-test p-values in brackets. Additional coefficients are available upon request. *** Significant at the 99.9% level; ** significant at the 99% level; * significant at the 95% level

**Table A4:** Reduced sample estimates

	PAID WORK		UNPAID WORK		CHILDCARE		LEISURE	
	**Women**	**Men**	**Women**	**Men**	**Women**	**Men**	**Women**	**Men**
	**(1)**	**(2)**	**(3)**	**(4)**	**(5)**	**(6)**	**(7)**	**(8)**
**UNITED KINGDOM**								
With the spouse	-0.251	0.241	0.355*	0.204	-0.115	-0.066	0.263**	0.308***
	(0.442)	(0.457)	(0.145)	(0.150)	(0.132)	(0.224)	(0.094)	(0.087)
	[0.572]	[0.598]	[0.015]	[0.176]	[0.385]	[0.768]	[0.005]	[0.000]
With children	1.003**	-3.538***	0.185	0.089	-	-	0.072	0.194
	(0.330)	(0.761)	(0.176)	(0.212)			(0.125)	(0.158)
	[0.003]	[0.000]	[0.295]	[0.674]			[0.566]	[0.222]
With others (family unit)	-0.050	0.220	0.459***	-0.040	0.292	0.107	0.032	-0.049
	(0.210)	(0.431)	(0.129)	(0.156)	(0.201)	(0.312)	(0.151)	(0.104)
	[0.812]	[0.611]	[0.000]	[0.798]	[0.150]	[0.732]	[0.832]	[0.637]
With others (non-fam. unit)	0.356*	0.015	0.649***	0.346	0.336	0.418	0.167	0.279**
	(0.139)	(0.162)	(0.144)	(0.180)	(0.208)	(0.355)	(0.085)	(0.098)
	[0.011]	[0.928]	[0.000]	[0.055]	[0.110]	[0.242]	[0.051]	[0.005]
All other controls	Yes	Yes	Yes	Yes	Yes	Yes	Yes	Yes
Observations	1,342	1,521	2,831	1,533	527	295	3,253	3,002
R-squared	0.067	0.124	0.089	0.061	0.223	0.263	0.048	0.077
Adjusted R-squared	0.053	0.112	0.082	0.048	0.194	0.210	0.042	0.071
F test *p*-value	< 0.001	< 0.001	< 0.001	< 0.001	< 0.001	< 0.001	< 0.001	< 0.001
AIC	4359.6	5235.4	10407.3	5351.8	1652.7	909.3	11107.4	9766.9
BIC	4468.9	5347.3	10538.1	5469.2	1738.0	983.0	11241.4	9899.1
**UNITED STATES**								
With the spouse	0.192	0.100	0.237*	0.408**	0.253*	0.345**	0.064	0.367***
	(0.262)	(0.297)	(0.116)	(0.126)	(0.112)	(0.112)	(0.124)	(0.088)
	[0.463]	[0.737]	[0.042]	[0.001]	[0.024]	[0.002]	[0.604]	[0.000]
With children	0.128	-0.370	0.377**	0.049	-	-	0.354***	0.349***
	(0.230)	(0.237)	(0.119)	(0.157)			(0.086)	(0.079)
	[0.579]	[0.118]	[0.002]	[0.755]			[0.000]	[0.000]
With others (family unit)	0.114	1.694***	0.111	0.038	0.433*	-0.575	0.354	0.284
	(0.439)	(0.437)	(0.244)	(0.369)	(0.173)	(0.441)	(0.184)	(0.169)
	[0.795]	[0.000]	[0.650]	[0.918]	[0.013]	[0.193]	[0.055]	[0.092]
With others (non-fam. unit)	0.091	-0.027	0.544***	0.452*	0.151	0.321	0.330***	0.193*
	(0.118)	(0.124)	(0.144)	(0.210)	(0.182)	(0.248)	(0.089)	(0.089)
	[0.442]	[0.827]	[0.000]	[0.031]	[0.408]	[0.197]	[0.000]	[0.030]
All other controls	Yes	Yes	Yes	Yes	Yes	Yes	Yes	Yes
Observations	1,820	2,140	3,599	1,912	1,449	961	4,805	5,191
R-squared	0.073	0.037	0.063	0.075	0.085	0.096	0.063	0.062
Adjusted R-squared	0.062	0.028	0.057	0.064	0.072	0.077	0.059	0.059
F test *p*-value	< 0.001	< 0.001	< 0.001	< 0.001	< 0.001	< 0.001	< 0.001	< 0.001
AIC	6576.7	7962.6	13480.8	6926.4	4762.1	2982.3	16529.0	18001.0
BIC	6697.9	8087.3	13616.9	7048.6	4873.0	3084.5	16671.5	18145.2

Note: The samples (UKTUS 2014–2015; ATUS SWB module 2010-2012-2013) are restricted to market work, leisure, housework, and childcare episodes of individuals between 21 and 65 years old. The dependent variable is the subjective enjoyment of episodes, which takes values from 1 (“not at all”) to 7 (“very much”), or the affective results of episodes, which take values from 0 (“not at all”) to 6 (“very much”). Robust standard errors, clustered at the individual level, in parentheses. T-test p-values in brackets. Additional coefficients are available upon request. *** Significant at the 99.9% level; ** significant at the 99% level; * significant at the 95% level

**Table A5:** Pooled sample and gender score estimates

	UNITED KINGDOM				UNITED STATES			
	**Paid work**	**Unpaid work**	**Child- care**	**Leisure**	**Paid work**	**Unpaid work**	**Child- care**	**Leisure**
	**(1)**	**(2)**	**(3)**	**(4)**	**(5)**	**(6)**	**(7)**	**(8)**
**UNITED KINGDOM**								
With the spouse	-0.870	0.348**	-0.089	0.250**	0.011	0.279**	0.205	0.109
	(0.449)	(0.132)	(0.169)	(0.085)	(0.257)	(0.107)	(0.113)	(0.105)
	[0.053]	[0.008]	[0.598]	[0.003]	[0.967]	[0.009]	[0.071]	[0.299]
With children	1.188***	0.161	-	0.188	0.510*	0.292**	-	0.378***
	(0.300)	(0.167)		(0.126)	(0.198)	(0.107)		(0.075)
	[0.000]	[0.335]		[0.136]	[0.010]	[0.006]		[0.000]
With others (family unit)	-0.199	0.361*	0.257	0.085	0.676	0.181	0.545***	0.238
	(0.267)	(0.145)	(0.201)	(0.138)	(0.496)	(0.211)	(0.156)	(0.182)
	[0.457]	[0.013]	[0.203]	[0.536]	[0.173]	[0.390]	[0.001]	[0.191]
With others (non-fam. unit)	0.398*	0.595***	0.383	0.261**	0.251*	0.578***	0.283	0.315***
	(0.155)	(0.137)	(0.206)	(0.096)	(0.107)	(0.127)	(0.180)	(0.086)
	[0.011]	[0.000]	[0.064]	[0.007]	[0.019]	[0.000]	[0.116]	[0.000]
Being male	-0.115	0.190	0.445	-0.233*	0.118	-0.120	0.277**	-0.203*
	(0.201)	(0.122)	(0.260)	(0.095)	(0.116)	(0.102)	(0.105)	(0.095)
	[0.570]	[0.118]	[0.088]	[0.015]	[0.310]	[0.240]	[0.008]	[0.032]
Being male X								
With the spouse	1.104	-0.183	-0.156	0.089	-0.334	0.170	0.048	0.279*
	(0.584)	(0.185)	(0.278)	(0.118)	(0.509)	(0.152)	(0.145)	(0.119)
	[0.059]	[0.325]	[0.576]	[0.453]	[0.512]	[0.263]	[0.742]	[0.019]
With children	-4.460***	0.039	-	-0.068	-0.512	-0.150	-	-0.038
	(0.794)	(0.256)		(0.200)	(0.290)	(0.168)		(0.100)
	[0.000]	[0.878]		[0.733]	[0.078]	[0.374]		[0.706]
With others (family unit)	0.421	-0.304	-0.269	-0.136	1.121	0.123	-1.055*	0.069
	(0.474)	(0.198)	(0.329)	(0.160)	(0.590)	(0.386)	(0.502)	(0.226)
	[0.374]	[0.126]	[0.413]	[0.394]	[0.057]	[0.751]	[0.036]	[0.759]
With others (non-fam. unit)	-0.398	-0.263	0.057	-0.004	-0.220	-0.057	-0.129	-0.122
	(0.213)	(0.230)	(0.402)	(0.137)	(0.136)	(0.205)	(0.258)	(0.115)
	[0.062]	[0.252]	[0.888]	[0.979]	[0.106]	[0.781]	[0.616]	[0.287]
All other controls	Yes	Yes	Yes	Yes	Yes	Yes	Yes	Yes
Observations	3,239	4,723	943	6,637	5,544	6,458	2,918	11,297
R-squared	0.093	0.064	0.175	0.068	0.040	0.055	0.079	0.066
Adjusted R-squared	0.086	0.058	0.153	0.065	0.035	0.051	0.071	0.064
F test *p*-value	< 0.001	< 0.001	< 0.001	< 0.001	< 0.001	< 0.001	< 0.001	< 0.001
AIC	11083.0	17127.8	3114.3	22200.4	20368.9	23982.0	9620.9	39023.4
BIC	11247.2	17302.3	3235.5	22384.0	20547.6	24164.9	9770.4	39221.3

Note: The samples (UKTUS 2014–2015; ATUS SWB module 2010-2012-2013) are restricted to market work, leisure, housework, and childcare episodes of individuals between 21 and 65 years old. The dependent variable is the subjective enjoyment of episodes, which takes values from 1 (“not at all”) to 7 (“very much”), or the affective results of episodes, which take values from 0 (“not at all”) to 6 (“very much”). Robust standard errors, clustered at the individual level, in parentheses. T-test p-values in brackets. Additional coefficients are available upon request. *** Significant at the 99.9% level; ** significant at the 99% level; * significant at the 95% level

**Table A6:** Estimates using standard errors

	PAID WORK		UNPAID WORK		CHILDCARE		LEISURE	
	**Women**	**Men**	**Women**	**Men**	**Women**	**Men**	**Women**	**Men**
	**(1)**	**(2)**	**(3)**	**(4)**	**(5)**	**(6)**	**(7)**	**(8)**
**UNITED KINGDOM**								
With the spouse	-0.730**	0.211	0.346***	0.102	-0.176	-0.048	0.291***	0.290***
	(0.282)	(0.246)	(0.068)	(0.084)	(0.116)	(0.147)	(0.057)	(0.053)
	[0.010]	[0.391]	[0.000]	[0.225]	[0.130]	[0.744]	[0.000]	[0.000]
With children	0.994*	-3.457***	0.152	0.111	-	-	0.107	0.214*
	(0.414)	(0.424)	(0.091)	(0.128)			(0.090)	(0.084)
	[0.016]	[0.000]	[0.096]	[0.385]			[0.234]	[0.011]
With others (family unit)	-0.149	0.230	0.419***	-0.044	0.155	0.150	0.038	-0.010
	(0.157)	(0.157)	(0.076)	(0.099)	(0.144)	(0.219)	(0.063)	(0.064)
	[0.340]	[0.142]	[0.000]	[0.656]	[0.281]	[0.493]	[0.547]	[0.874]
With others (non-fam. unit)	0.358***	0.029	0.619***	0.389**	0.470*	0.490	0.217**	0.296***
	(0.076)	(0.077)	(0.101)	(0.144)	(0.194)	(0.360)	(0.066)	(0.069)
	[0.000]	[0.710]	[0.000]	[0.007]	[0.016]	[0.175]	[0.001]	[0.000]
All other controls	Yes	Yes	Yes	Yes	Yes	Yes	Yes	Yes
Observations	1,501	1,738	3,047	1,676	618	325	3,446	3,191
R-squared	0.099	0.117	0.090	0.056	0.207	0.288	0.065	0.081
Adjusted R-squared	0.087	0.106	0.084	0.044	0.182	0.241	0.060	0.075
F test *p*-value	< 0.001	< 0.001	< 0.001	< 0.001	< 0.001	< 0.001	< 0.001	< 0.001
AIC	4919.2	6037.9	11196.9	5865.5	2049.7	1019.8	11741.2	10423.6
BIC	5030.8	6158.0	11329.4	5984.8	2138.2	1099.3	11876.4	10557.1
**UNITED STATES**								
With the spouse	0.195	-0.417	0.267***	0.422***	0.184*	0.324***	0.113*	0.378***
	(0.275)	(0.237)	(0.065)	(0.079)	(0.081)	(0.076)	(0.048)	(0.050)
	[0.478]	[0.079]	[0.000]	[0.000]	[0.023]	[0.000]	[0.020]	[0.000]
With children	0.360	-0.006	0.338***	0.048	-	-	0.373***	0.361***
	(0.267)	(0.355)	(0.068)	(0.104)			(0.050)	(0.060)
	[0.178]	[0.986]	[0.000]	[0.643]			[0.000]	[0.000]
With others (family unit)	0.615	1.748	0.168	0.120	0.555***	-0.541**	0.333***	0.277**
	(0.431)	(0.915)	(0.120)	(0.180)	(0.154)	(0.182)	(0.073)	(0.085)
	[0.153]	[0.056]	[0.163]	[0.506]	[0.000]	[0.003]	[0.000]	[0.001]
With others (non-fam. unit)	0.144	0.102	0.553***	0.511***	0.258*	0.252	0.343***	0.170***
	(0.084)	(0.072)	(0.097)	(0.130)	(0.104)	(0.154)	(0.047)	(0.045)
	[0.086]	[0.157]	[0.000]	[0.000]	[0.013]	[0.102]	[0.000]	[0.000]
All other controls	Yes	Yes	Yes	Yes	Yes	Yes	Yes	Yes
Observations	2,432	3,112	4,187	2,271	1,778	1,140	5,347	5,950
R-squared	0.086	0.029	0.056	0.076	0.083	0.084	0.063	0.067
Adjusted R-squared	0.078	0.022	0.052	0.068	0.073	0.068	0.060	0.064
F test *p*-value	< 0.001	< 0.001	< 0.001	< 0.001	< 0.001	< 0.001	< 0.001	< 0.001
AIC	8785.3	11528.1	15643.2	8300.4	6049.7	3551.1	18355.4	20644.3
BIC	8912.8	11661.0	15782.7	8426.4	6164.8	3657.0	18500.3	20791.5

Note: The samples (UKTUS 2014–2015; ATUS SWB module 2010-2012-2013) are restricted to market work, leisure, housework, and childcare episodes of individuals between 21 and 65 years old. The dependent variable is the subjective enjoyment of episodes, which takes values from 1 (“not at all”) to 7 (“very much”), or the affective results of episodes, which take values from 0 (“not at all”) to 6 (“very much”). Standard errors in parentheses. T-test p-values in brackets. Additional coefficients are available upon request. *** Significant at the 99.9% level; ** significant at the 99% level; * significant at the 95% level

**Table A7:** Size effects of main variables

	PAID WORK		UNPAID WORK		CHILDCARE		LEISURE	
	**Women**	**Men**	**Women**	**Men**	**Women**	**Men**	**Women**	**Men**
	**(1)**	**(2)**	**(3)**	**(4)**	**(5)**	**(6)**	**(7)**	**(8)**
**UNITED KINGDOM**								
With the spouse	0.005	0.000	0.009	0.001	0.004	0.000	0.008	0.009
	(0.045)	(0.003)	(0.096)	(0.016)	(0.018)	(0.001)	(0.115)	(0.116)
With children	0.004	0.037	0.001	0.001	-	-	0.004	0.002
	(0.039)	(0.318)	(0.010)	(0.009)	-	-	(0.063)	(0.026)
With others (family unit)	0.001	0.001	0.010	0.000	0.002	0.002	0.000	0.000
	(0.006)	(0.011)	(0.110)	(0.002)	(0.009)	(0.005)	(0.002)	(0.008)
With others (non-fam. unit)	0.015	0.000	0.012	0.004	0.010	0.006	0.003	0.006
	(0.150)	(0.001)	(0.137)	(0.078)	(0.047)	(0.021)	(0.049)	(0.073)
Model size	0.099	0.117	0.090	0.056	0.207	0.288	0.065	0.081
All other controls	Yes	Yes	Yes	Yes	Yes	Yes	Yes	Yes
Observations	1,501	1,738	3,047	1,676	618	325	3,446	3,191
**UNITED STATES**								
With the spouse	0.000	0.001	0.004	0.013	0.003	0.016	0.001	0.010
	(0.002)	(0.035)	(0.071)	(0.165)	(0.035)	(0.193)	(0.016)	(0.142)
With children	0.001	0.000	0.006	0.000	-	-	0.011	0.006
	(0.009)	(0.009)	(0.107)	(0.001)	-	-	(0.166)	(0.093)
With others (family unit)	0.001	0.001	0.001	0.000	0.007	0.008	0.004	0.002
	(0.009)	(0.042)	(0.009)	(0.003)	(0.089)	(0.093)	(0.062)	(0.027)
With others (non-fam. unit)	0.001	0.001	0.008	0.007	0.004	0.002	0.010	0.002
	(0.014)	(0.021)	(0.137)	(0.089)	(0.042)	(0.029)	(0.155)	(0.036)
Model size	0.086	0.029	0.056	0.076	0.084	0.084	0.063	0.067
All other controls	Yes	Yes	Yes	Yes	Yes	Yes	Yes	Yes
Observations	2,432	3,112	4,187	2,271	1,778	1,140	5,347	5,950

Note: The samples (UKTUS 2014–2015; ATUS SWB module 2010-2012-2013) are restricted to market work, leisure, housework, and childcare episodes of individuals between 21 and 65 years old. The dependent variable is the subjective enjoyment of episodes, which takes values from 1 (“not at all”) to 7 (“very much”), or the affective results of episodes, which take values from 0 (“not at all”) to 6 (“very much”). Size effects (eta squared) computed from estimates in Table A8. Relative size effects (i.e., variable size effect, over model size) in parentheses

**Table A8:** Estimates of additional coefficients - United Kingdom

VARIABLES	PAID WORK		UNPAID WORK		CHILDCARE		LEISURE	
	**Women**	**Men**	**Women**	**Men**	**Women**	**Men**	**Women**	**Men**
	**(1)**	**(2)**	**(3)**	**(4)**	**(5)**	**(6)**	**(7)**	**(8)**
Age	0.007	-0.011	-0.001	0.016*	-0.080***	-0.031	-0.003	0.001
	(0.010)	(0.013)	(0.006)	(0.006)	(0.018)	(0.018)	(0.005)	(0.005)
	[0.493]	[0.386]	[0.824]	[0.010]	[0.000]	[0.081]	[0.564]	[0.899]
Educ.: Secondary	0.232	0.134	0.528*	-0.291	-1.634**	-0.931	0.386	0.356
	(0.334)	(0.662)	(0.267)	(0.459)	(0.528)	(0.837)	(0.304)	(0.282)
	[0.487]	[0.840]	[0.049]	[0.526]	[0.002]	[0.269]	[0.206]	[0.208]
Educ.: University	0.255	0.182	0.503*	-0.263	-1.515***	-0.266	0.399	0.145
	(0.285)	(0.658)	(0.251)	(0.455)	(0.431)	(0.794)	(0.286)	(0.283)
	[0.371]	[0.782]	[0.046]	[0.563]	[0.001]	[0.739]	[0.164]	[0.609]
Being Hispanic	-0.057	-0.218	-0.300	0.101	0.904	-0.035	-0.204	0.189
	(0.291)	(0.373)	(0.231)	(0.203)	(0.593)	(0.364)	(0.172)	(0.150)
	[0.844]	[0.560]	[0.195]	[0.619]	[0.130]	[0.923]	[0.236]	[0.208]
Living in couple	-0.157	-0.320	-0.204	-0.414*	-0.638*	-2.172**	-0.330**	-0.268*
	(0.209)	(0.271)	(0.157)	(0.177)	(0.247)	(0.678)	(0.112)	(0.126)
	[0.453]	[0.240]	[0.194]	[0.020]	[0.011]	[0.002]	[0.003]	[0.033]
Family size	-0.154	0.152	-0.145*	0.092	0.360***	-0.286	0.016	-0.016
	(0.091)	(0.095)	(0.067)	(0.074)	(0.105)	(0.206)	(0.052)	(0.050)
	[0.092]	[0.110]	[0.032]	[0.214]	[0.001]	[0.167]	[0.761]	[0.754]
Number of children	0.111	0.066	-0.114	-0.137	-0.438**	-0.078	0.015	-0.093
	(0.126)	(0.168)	(0.098)	(0.119)	(0.146)	(0.271)	(0.081)	(0.078)
	[0.379]	[0.694]	[0.245]	[0.251]	[0.003]	[0.774]	[0.856]	[0.235]
Employment: self-employed	-	1.104	-0.672	-0.515	-	1.209	-0.629*	-0.415
		(0.970)	(0.396)	(0.362)		(0.672)	(0.277)	(0.345)
		[0.256]	[0.090]	[0.156]		[0.076]	[0.024]	[0.230]
Full time worker	-0.168	0.403	0.018	0.066	0.077	0.973*	0.132	0.202
	(0.234)	(0.554)	(0.148)	(0.258)	(0.225)	(0.437)	(0.146)	(0.178)
	[0.474]	[0.468]	[0.905]	[0.798]	[0.733]	[0.029]	[0.368]	[0.257]
Weekday	0.109	-0.100	0.061	0.203	-0.341	-0.136	-0.139	0.050
	(0.201)	(0.251)	(0.138)	(0.163)	(0.217)	(0.236)	(0.148)	(0.140)
	[0.589]	[0.690]	[0.656]	[0.214]	[0.119]	[0.566]	[0.351]	[0.720]
Log-Market work time	0.214	-0.085	-	-	-	-	-	-
	(0.195)	(0.236)						
	[0.273]	[0.720]						
Log-Housework time	-	-	-0.093	-0.047	-	-	-	-
			(0.092)	(0.083)				
			[0.317]	[0.570]				
Log-Childcare time	-	-	-	-	-0.055	0.359**	-	-
					(0.125)	(0.126)		
					[0.657]	[0.006]		
Log-Leisure time	-	-	-	-	-	-	0.201*	0.097
							(0.079)	(0.106)
							[0.011]	[0.360]
Constant	3.296*	4.930**	5.416***	4.209***	8.164***	7.350***	4.498***	4.407***
	(1.300)	(1.654)	(0.589)	(0.688)	(1.466)	(1.197)	(0.556)	(0.870)
	[0.012]	[0.003]	[0.000]	[0.000]	[0.000]	[0.000]	[0.000]	[0.000]
Region F.E.	Yes	Yes	Yes	Yes	Yes	Yes	Yes	Yes
Observations	1,501	1,738	3,047	1,676	618	325	3,446	3,191

Note: The sample (UKTUS 2014–2015) is restricted to paid work, unpaid work, childcare and leisure episodes of individuals between 21 and 65 years old. The dependent variable is the subjective enjoyment of episodes, which takes values from 1 (“not at all”) to 7 (“very much”). Robust standard errors, clustered at the individual level, in parentheses. T-test p-values in brackets. *** Significant at the 99.9% level; ** significant at the 99% level; * significant at the 95% level

**Table A9:** Estimates of additional coefficients - United States

VARIABLES	PAID WORK		UNPAID WORK		CHILDCARE		LEISURE	
	**Women**	**Men**	**Women**	**Men**	**Women**	**Men**	**Women**	**Men**
	**(1)**	**(2)**	**(3)**	**(4)**	**(5)**	**(6)**	**(7)**	**(8)**
Age	0.014***	0.010*	0.001	0.007	-0.027***	-0.008	0.004	0.004
	(0.004)	(0.004)	(0.005)	(0.005)	(0.007)	(0.007)	(0.003)	(0.003)
	[0.000]	[0.013]	[0.840]	[0.136]	[0.000]	[0.257]	[0.207]	[0.269]
Educ.: Secondary	-0.314	-0.286	-0.643	-0.372	-0.742***	-0.390*	-0.058	-0.294
	(0.446)	(0.319)	(0.441)	(0.263)	(0.207)	(0.163)	(0.281)	(0.202)
	[0.482]	[0.370]	[0.145]	[0.157]	[0.000]	[0.017]	[0.837]	[0.146]
Educ.: University	-0.531	-0.472	-0.829	-0.484	-0.733***	-0.672***	-0.282	-0.379
	(0.445)	(0.320)	(0.444)	(0.280)	(0.215)	(0.181)	(0.284)	(0.204)
	[0.233]	[0.141]	[0.062]	[0.084]	[0.001]	[0.000]	[0.320]	[0.064]
Being Hispanic	0.171	-0.221*	-0.420***	-0.631***	-0.265	-0.276*	-0.034	-0.115
	(0.129)	(0.111)	(0.123)	(0.157)	(0.141)	(0.138)	(0.111)	(0.100)
	[0.188]	[0.047]	[0.001]	[0.000]	[0.061]	[0.046]	[0.757]	[0.249]
Living in couple	-0.052	0.006	0.000	-0.049	-0.078	0.040	0.056	0.049
	(0.092)	(0.103)	(0.107)	(0.157)	(0.145)	(0.176)	(0.089)	(0.097)
	[0.575]	[0.951]	[0.999]	[0.753]	[0.590]	[0.820]	[0.534]	[0.617]
Family size	0.091*	-0.026	-0.037	-0.015	-0.012	0.008	-0.102	-0.012
	(0.046)	(0.053)	(0.061)	(0.091)	(0.072)	(0.142)	(0.065)	(0.056)
	[0.047]	[0.623]	[0.545]	[0.867]	[0.864]	[0.953]	[0.119]	[0.831]
Number of children	0.053	0.040	-0.031	0.074	-0.055	0.015	0.094	-0.011
	(0.063)	(0.071)	(0.083)	(0.106)	(0.084)	(0.157)	(0.080)	(0.068)
	[0.398]	[0.576]	[0.705]	[0.485]	[0.512]	[0.924]	[0.236]	[0.871]
Employment: self-employed	0.387*	0.076	0.040	0.292	0.010	-0.178	0.257**	-0.155
	(0.151)	(0.132)	(0.152)	(0.176)	(0.170)	(0.167)	(0.091)	(0.099)
	[0.010]	[0.567]	[0.791]	[0.097]	[0.953]	[0.288]	[0.005]	[0.119]
Full time worker	-0.335**	0.027	0.165	-0.288	0.073	-0.139	0.124	-0.076
	(0.119)	(0.154)	(0.110)	(0.183)	(0.116)	(0.231)	(0.089)	(0.122)
	[0.005]	[0.860]	[0.132]	[0.116]	[0.528]	[0.548]	[0.166]	[0.531]
Weekday	-0.047	-0.046	-0.034	-0.102	0.024	0.081	-0.059	-0.106
	(0.118)	(0.103)	(0.094)	(0.128)	(0.103)	(0.099)	(0.084)	(0.080)
	[0.691]	[0.657]	[0.722]	[0.429]	[0.817]	[0.417]	[0.480]	[0.187]
Log-Market work time	-0.100	-0.099	-	-	-	-	-	-
	(0.113)	(0.132)						
	[0.376]	[0.456]						
Log-Housework time	-	-	-0.055	-0.005	-	-	-	-
			(0.065)	(0.076)				
			[0.399]	[0.953]				
Log-Childcare time	-	-	-	-	0.075	0.076	-	-
					(0.073)	(0.064)		
					[0.305]	[0.233]		
Log-Leisure time	-	-	-	-	-	-	0.136**	0.146**
							(0.049)	(0.050)
							[0.006]	[0.003]
Constant	4.514***	4.737***	5.350***	4.848***	5.877***	5.629***	3.876***	3.856***
	(0.790)	(0.820)	(0.699)	(0.643)	(0.562)	(0.653)	(0.434)	(0.405)
	[0.000]	[0.000]	[0.000]	[0.000]	[0.000]	[0.000]	[0.000]	[0.000]
Region F.E.	Yes	Yes	Yes	Yes	Yes	Yes	Yes	Yes
Observations	2,432	3,112	4,187	2,271	1,778	1,140	5,347	5,950

Note: The sample (ATUS SWB module 2010-2012-2013) is restricted to paid work, unpaid work, childcare and leisure episodes of individuals between 21 and 65 years old. The dependent variable is the happiness scale of episodes, which takes values from 0 (“not at all”) to 6 (“very much”). Robust standard errors, clustered at the individual level, in parentheses. T-test p-values in brackets. *** Significant at the 99.9% level; ** significant at the 99% level; * significant at the 95% level

## Footnotes

1 Affective well-being refers to how individuals feel during an activity, and can be measured by specific positive and negative emotional experiences, such as enjoyment, happiness, joy, anxiety, sadness, stress, and anger (Kahneman & Deaton, 2010). A related concept, developed in the economic discipline, is the notion of experienced utility, which refers to the experience of feelings and emotions resulting from the outcome of a choice. When experienced utility is measured in real time it is known as instant utility (Kahneman et al., 1997). Kahneman et al., (2004) and Kahneman & Krueger (2006) provide a seminal contribution in which instant utility is derived from time use data.

2 Experienced utility, or instantaneous well-being, refers to the instant subjective feelings (enjoyment, or happiness) of individuals when doing a specific activity. Thus, these measures attempt to capture “the moment-to-moment flow of pleasure or pain” (Kahneman & Krueger, 2006). It is important to distinguish these measures from other measures of subjective well-being, such as cognitive measures based on overall, or average, life satisfaction or subjective well-being. It is important to note that positive instant feelings while doing certain activities do not necessarily mean higher general life satisfaction, or higher subjective well-being. See Fritjers (2022) for a recent review.

3 Our focus on the UK and the US is because, to the best of our knowledge, there are no datasets with information on time use and enjoyment/happiness for other countries in a similar period. Thus, we cannot generalize results to other economies, especially those from outside the Western, Educated, Industrialized, Rich and Democratic (WEIRD) world (Henrich et al., 2010).

4 Furthermore, some national surveys have updated their scope to cover behaviors linked to COVID-19 confinements, such as the ATUS and the Panel Study of Income Dynamics in the US, and the YouGov data in the UK.

5 The measurement of individual well-being dates back to authors such as Francis Ysidro Edgeworth and Alfred Marshall (Kahneman & Krueger, 2006), and refers to how a person believes their lives are going (Diener et al., 2018). Today, the concept of individual subjective well-being has evolved and there exist several measures and methods to measure it (see Fritjers (2022) for a recent review).

6 Affective well-being refers to how individuals feel during an activity, and can be measured by specific positive and negative emotional experiences, such as enjoyment, happiness, joy, anxiety, sadness, stress, and anger (Kahneman & Deaton, 2010). A related concept, developed in the economic discipline, is the notion of experienced utility, which refers to the experience of feelings and emotions resulting from the outcome of a choice. When experienced utility is measured in real time it is known as instant utility (Kahneman et al., 1997), Kahneman et al., (2004), and Kahneman & Krueger (2006) provide a seminal contribution in which instant utility is derived from time use data.

7 Both the UKTUS and the ATUS data are free access from the UK Data Service, and the Bureau of Labor Statistics, respectively. Data can also be freely downloaded from the Integrated Public Use Microdata Series (IPUMS) online system (https://timeuse.ipums.org/). The replication files for sample construction, final samples, and replication files to run the empirical analyses are available at https://bit.ly/3sh8Air.

8 A detailed list of activities and classifications is available upon request.

9 See Sevilla et al., (2012) for a review of the different methods in the literature to assess feelings during time use activities.

10 All the statistics and results are computed using specific weights computed at the activity level.

11 It is beyond the scope of this manuscript to analyze gender differences in the average time devoted to the four time use categories, given that much prior research has documented gender differences in the uses of time. Results are available upon request.

12 We also run estimates including an interaction term between the vector of dummy variables ${{\rm{J}}_{{\rm{ij}}}}$, and the dummy that identifies episodes taking place at home, in order to capture whether there is an additional correlation between joint activities and the experienced satisfaction, if those activities take place at home, or elsewhere. These interactions were not statistically significant, indicating that such an additional effect is not relevant at standard levels. Interaction estimates are available upon request.

13 Among the socio-demographic characteristics included as explanatory variables, we include the (log) total time devoted to the activity of reference by the individual. Following Hamermesh (2020), the total time devoted to paid work is reduced by one third, which is spent in more leisure.

14 See https://www.bls.gov/tus/covid19.htm.

## References

[CR1] Adams-Prassl, A. (2020). The gender wage gap on an online labour market: The cost of interruptions. CEPR Discussion paper No. DP14294

[CR2] Aguiar M, Hurst E (2007). Measuring trends in leisure: The allocation of time over five decades. Quarterly Journal of Economics.

[CR3] Aguiar M, Hurst E, Karabarbounis L (2013). Time use during the great recession. American Economic Review.

[CR4] Beblo M, Robledo JR (2008). The wage gap and the leisure gap for double-earner couples. Journal of Population Economics.

[CR5] Berik G, Kongar E (2013). Time allocation of married mothers and fathers in hard times: The 2007–09 US recession. Feminist Economics.

[CR6] Billor N, Hadi AS, Velleman PF (2000). BACON: blocked adaptive computationally efficient outlier nominators. Computational Statistics & Data Analysis.

[CR7] Boll, C., Müller, D., & Schüller, S. (2021). Neither backlash nor convergence: dynamics of intracouple childcare division after the first COVID-19 lockdown and subsequent reopening in Germany. CESifo Working Paper No. 9091

[CR8] Brand, R., Timme, S., & Nosrat, S. (2020). When pandemic hits: exercise frequency and subjective well-being during COVID-19 pandemic. *Frontiers in Psychology*, 2391. 10.3389/fpsyg.2020.570567 10.3389/fpsyg.2020.570567 PMC7541696 33071902

[CR9] Brindal, E., Ryan, J. C., Kakoschke, N., Golley, S., Zajac, I. T., & Wiggins, B. (2021). Individual differences and changes in lifestyle behaviours predict decreased subjective well-being during COVID-19 restrictions in an Australian sample. *Journal of Public Health*, advanced online publication. 10.1093/pubmed/fdab040 10.1093/pubmed/fdab040 PMC7989396 33683320

[CR10] Carlson, D. L., Petts, R. J., & Pepin, J. R. (2021). Changes in US Parents’ Domestic Labor During the Early Days of the COVID-19 Pandemic. *Sociological Inquiry*, advanced online publication. 10.1111/soin.12459 10.1111/soin.12459 PMC8662176 34908600

[CR11] Carmichael F, Hulme C, Sheppard S, Connell G (2008). Work–life imbalance: Informal care and paid employment in the UK. Feminist Economics.

[CR12] Cortina JM (1993). What is coefficient alpha? An examination of theory and applications. Journal of Applied Psychology.

[CR13] Cosaert, S., Theloudis, A., & Verheyden, B. (2022). Togetherness in the Household. *American Economic Journal-Microeconomics*, advanced online publication. https://www.aeaweb.org

[CR14] Craig L, Churchill B (2021). Working and caring at home: Gender differences in the effects of COVID-19 on paid and unpaid labor in Australia. Feminist Economics.

[CR15] Del Boca D, Oggero N, Profeta P, Rossi M (2020). Women’s and men’s work, housework and childcare, before and during COVID-19. Review of Economics of the Household.

[CR16] Diener E, Lucas RE, Oishi S, Hall N, Donnellan MB (2018). Advances and open questions in the science of subjective well-being. Collabra: Psychology.

[CR17] Eaton SC (2005). Eldercare in the United States: Inadequate, inequitable, but not a lost cause. Feminist Economics.

[CR18] Farré, L., Fawaz, Y., González, L., & Graves, J. (2020). How the COVID-19 lockdown affected gender inequality in paid and unpaid work in Spain. IZA Discussion Paper No. 13434

[CR19] Ferrer-i‐Carbonell A, Frijters P (2004). How important is methodology for the estimates of the determinants of happiness?. The Economic Journal.

[CR20] Foa, R., Gilbert, S., & Fabian, M. O. (2020). COVID-19 and subjective well-being: Separating the effects of lockdowns from the pandemic. *The Lancet Psychiatry*, advanced online publication. 10.2139/ssrn.3674080

[CR21] Fritjers, P. (2022). Measuring subjective wellbeing. In Zimmermann, K.F. (Ed.), *Handbook of Labor, Human Resources and Population Economics*, advanced online publication. 10.1007/978-3-319-57365-6_189-1

[CR22] Fujiwara, D., Dolan, P., Lawton, R., Behzadnejad, F., Lagarde, A., Maxwell, C., & Peytrignet, S. (2020). *Wellbeing costs of COVID-19 in the UK*. Report to the World Health Organization

[CR23] Gershuny J (2013). National utility: Measuring the enjoyment of activities. European Sociological Review.

[CR24] Gershuny J, Halpin B, Offer A (1996). Time use, quality of life and process benefits. Pursuit of the Quality of Life.

[CR25] Gimenez-Nadal JI, Molina JA (2014). Regional unemployment, gender, and time allocation of the unemployed. Review of Economics of the Household.

[CR26] Gimenez-Nadal JI, Molina JA (2015). Voluntary activities and daily happiness in the United States. Economic Inquiry.

[CR27] Giménez-Nadal JI, Molina JA, Velilla J (2020). Work time and well-being for workers at home: evidence from the American Time Use Survey. International Journal of Manpower.

[CR28] Giménez-Nadal JI, Molina JA, Velilla J (2021). Two-way commuting: Asymmetries from time use surveys. Journal of Transport Geography.

[CR29] Gimenez-Nadal JI, Sevilla A (2011). The time-crunch paradox. Social Indicators Research.

[CR30] Gimenez-Nadal JI, Sevilla A (2012). Trends in time allocation: A cross-country analysis. European Economic Review.

[CR31] Hallberg D (2003). Synchronous leisure, jointness and household labor supply. Labour Economics.

[CR32] Hamermesh DS (2020). Life satisfaction, loneliness and togetherness, with an application to Covid-19 lock-downs. Review of Economics of the Household.

[CR33] Hamermesh DS, Stancanelli E (2015). Long workweeks and strange hours. ILR Review.

[CR34] Helliwell, J. F., & Putnam, R. D. (2005). The social context of well-being. In F. Huppert, Beylis, N., & Keverne, B. (Eds), *The Science of Well-Being*, ch. 17. Oxford University Press

[CR35] Henrich J, Heine SJ, Norenzayan A (2010). The weirdest people in the world?. Behavioral and Brain Sciences.

[CR36] Hoang TTA, Knabe A (2021). Time use, unemployment, and well-being: an empirical analysis using British time-use data. Journal of Happiness Studies.

[CR37] James G, Witten D, Hastie T, Tibshirani R (2013). An introduction to statistical learning.

[CR38] Jenkins, S. P., & Osberg, L. (2004). Nobody to play with? The implications of leisure coordination. In D. Hamermesh, & G. Pfann (Eds.), *The Economics of Time Use*. Elsevier

[CR39] Juster FT, Stafford FP (1985). Time, Goods, and Well-Being.

[CR40] Kahneman D, Krueger AB, Schkade DA, Schwarz N, Stone AA (2004). A survey method for characterizing daily life experience: The day reconstruction method. Science.

[CR41] Kahneman, D., & Deaton, A. (2010). High income improves evaluation of life but not emotional well-being. *Proceedings of the National Academy of Sciences*, 107(38), 16489–16493. 10.1073/pnas.1011492107 10.1073/pnas.1011492107 PMC2944762 20823223

[CR42] Kahneman D, Krueger AB (2006). Developments in the measurement of subjective well-being. Journal of Economic Perspectives.

[CR43] Knabe A, Rätzel S, Schöb R, Weimann J (2010). Dissatisfied with life but having a good day: time-use and well‐being of the unemployed. The Economic Journal.

[CR44] Kreyenfeld M, Zinn S (2021). Coronavirus and care. Demographic Research.

[CR45] Krueger AB (2007). Are we having more fun yet? Categorizing and evaluating changes in time allocation. Brookings Papers on Economic Activity.

[CR46] Krueger AB (2009). Measuring the Subjective Well-Being of Nations: National Accounts of Time Use and Well-Being.

[CR47] Long TQ (2021). Individual subjective well-being during the COVID-19 pandemic. Sustainability.

[CR48] Möhring K, Naumann E, Reifenscheid M, Wenz A, Rettig T, Krieger U, Blom AG (2021). The COVID-19 pandemic and subjective well-being: longitudinal evidence on satisfaction with work and family. European Societies.

[CR49] Qian Y, Fan W (2019). Men and women at work: Occupational gender composition and affective well-being in the United States. Journal of Happiness Studies.

[CR50] Recchi E, Ferragina E, Helmeid E, Pauly S, Safi M, Sauger N, Schradie J (2020). The “eye of the hurricane” paradox: an unexpected and unequal rise of well-being during the Covid-19 lockdown in France. Research in Social Stratification and Mobility.

[CR51] Ruiz MC, Devonport TJ, Chen-Wilson CHJ, Nicholls W, Cagas JY, Fernandez-Montalvo J, Robazza C (2021). A cross-cultural exploratory study of health behaviors and wellbeing during COVID-19. Frontiers in Psychology.

[CR52] Sevilla A, Gimenez-Nadal JI, Gershuny J (2012). Leisure inequality in the United States: 1965–2003. Demography.

[CR53] Sevilla A, Smith S (2020). Baby steps: The gender division of childcare during the COVID-19 pandemic. Oxford Review of Economic Policy.

[CR54] Stiglitz, J. E., Sen, A., & Fitoussi, J. P. (2009). Report by the Commission on the Measurement of Economic Performance and Social Progress. https://www.oecd.org/statistics/measuring-economic-social-progress/

[CR55] Sullivan O (1996). Time co-ordination, the domestic division of labour and affective relations: Time use and the enjoyment of activities within couples. Sociology.

[CR56] Sullivan O (1996). The enjoyment of activities: Do couples affect each others’ well-being?. Social Indicators Research.

[CR57] Szalai A (1972). The Use of Time.

[CR58] Taber KS (2018). The use of Cronbach’s alpha when developing and reporting research instruments in science education. Research in Science Education.

[CR59] Watson D, Clark LA, Tellegen A (1988). Development and validation of brief measures of positive and negative affect: the PANAS scales. Journal of Personality and Social Psychology.

[CR60] Yaish M, Mandel H, Kristal T (2021). Has the economic lockdown following the Covid-19 pandemic changed the gender division of labor in Israel?. Gender & Society.

[CR61] Yerkes MA, André SC, Besamusca JW, Kruyen PM, Remery CL, van der Zwan R, Geurts SA (2020). ‘Intelligent’lockdown, intelligent effects? Results from a survey on gender (in) equality in paid work, the division of childcare and household work, and quality of life among parents in the Netherlands during the Covid-19 lockdown. PloS One.

[CR62] Zacher H, Rudolph CW (2021). Individual differences and changes in subjective wellbeing during the early stages of the COVID-19 pandemic. American Psychologist.

